# Comparative and Functional Analyses of Two Sequenced *Paenibacillus polymyxa* Genomes Provides Insights Into Their Potential Genes Related to Plant Growth-Promoting Features and Biocontrol Mechanisms

**DOI:** 10.3389/fgene.2020.564939

**Published:** 2020-12-17

**Authors:** Jin-Yi Li, Tan-Tan Gao, Qi Wang

**Affiliations:** ^1^MOA Key Lab of Pest Monitoring and Green Management, Department of Plant Pathology, College of Plant Protection, China Agricultural University, Beijing, China; ^2^College of Life Science and Technology, Beijing University of Chemical Technology, Beijing, China; ^3^Key Laboratory for Northern Urban Agriculture, Ministry of Agriculture and Rural Affairs, Beijing University of Agriculture, Beijing, China

**Keywords:** genome sequencing, *Paenibacillus polymyxa*, plant growth-promoting, secondary metabolites, taxonomic position, inhibitory activity

## Abstract

Many bacteria belonging to *Paenibacillus polymyxa* are plant growth-promoting rhizobacteria (PGPR) with the potential to promote plant growth and suppress phytopathogens and have been used as biological control agents (BCAs). However, the growth promotion and biocontrol mechanisms of *P. polymyxa* have not been thoroughly elucidated thus far. In this investigation, the genome sequences of two *P. polymyxa* strains, ZF129 and ZF197, with broad anti-pathogen activities and potential for growth promotion were comparatively studied. Comparative and functional analyses of the two sequenced *P. polymyxa* genomes showed that the ZF129 genome consists of one 5,703,931 bp circular chromosome and two 79,020 bp and 37,602 bp plasmids, designated pAP1 and pAP2, respectively. The complete genome sequence of ZF197 consists of one 5,507,169 bp circular chromosome and one 32,065 bp plasmid, designated pAP197. Phylogenetic analysis revealed that ZF129 is highly similar to two *P. polymyxa* strains, HY96-2 and SQR-21, while ZF197 is highly similar to *P. polymyxa* strain J. The genes responsible for secondary metabolite synthesis, plant growth-promoting traits, and systemic resistance inducer production were compared between strains ZF129 and ZF197 as well as other *P. polymyxa* strains. The results indicated that the variation of the corresponding genes or gene clusters between strains ZF129 and ZF197 may lead to different antagonistic activities of their volatiles or cell-free supernatants against *Fusarium oxysporum*. This work indicates that plant growth promotion by *P. polymyxa* is largely mediated by phytohormone production, increased nutrient availability and biocontrol mechanisms. This study provides an in-depth understanding of the genome architecture of *P. polymyxa*, revealing great potential for the application of this bacterium in the fields of agriculture and horticulture as a PGPR.

## Introduction

Plant growth-promoting rhizobacteria (PGPR) have been identified as environmentally friendly alternatives to traditional agrochemicals for improving crop yield and quality (Kloepper et al., 1980). *Paenibacillus polymyxa* (formerly *Bacillus polymyxa*), which is an important beneficial member of the PGPR, has been applied in the fields of agriculture and horticulture in the form of soil inoculants to control a wide array of plant pathogens (Raza et al., 2008). *P. polymyxa* is a prominent biofertilizer and biocontrol agent that has been reported to suppress a wide variety of fungal and bacterial plant diseases, such as those caused by the fungi *Rhizoctonia solani*, *Fusarium oxysporum*, *Botrytis cinereal*, *Phytophthora parasitica* var. *nicotianae*, and *Penicillium digitatum* (Chen et al., 2011; Lai et al., 2012; Ren et al., 2012; Weselowski et al., 2016; Liu et al., 2018) and the bacteria *Pseudomonas syringae* pv. *tomato*, *P. syringae* pv. *lachrymans, Xanthomonas campestris*, *X. oryzae* pv. *oryzae*, *Acidovorax avenae* subsp. *citrulli*, and *Ralstonia solanacearum* (Li et al., 2010; Shi et al., 2012; Weselowski et al., 2016; Abdallah et al., 2019). Although the suppression of plant diseases by *P. polymyxa* strains has been continuously reported, further research into their detailed biocontrol mechanisms, especially at the molecular level, is necessary.

Plant growth-promoting rhizobacteria stably colonize the plant rhizosphere and enhance plant growth due to their capacities for phytohormone production, phosphate solubilization, nitrogen fixation, and antibiotic biosynthesis (Xie et al., 2016). *P. polymyxa* has been reported to produce various potent antimicrobial and volatile compounds that reduce plant disease severity (Eastman et al., 2014), such as antifungal and antibacterial metabolites (Zhao et al., 2011; Mageshwaran et al., 2012; Raza et al., 2015; Liu et al., 2018), thereby promoting growth (Anand et al., 2013; Padda et al., 2016) and inducing plant defenses (Mei et al., 2014; Shi et al., 2017; Luo et al., 2018). The types and amounts of antimicrobial substances generated by a beneficial bacterium affect its antimicrobial spectra and biocontrol efficacy. *P. polymyxa* can produce several kinds of antibiotic compounds, that can suppress the growth of pathogens under both laboratory and field conditions, including polymyxins and antifungal compounds such as fusaricidin (Choi et al., 2009; Padda et al., 2017; Liu et al., 2018). Moreover, it has been reported that *P. polymyxa* secretes other types of antibiotics, such as 1-octen-3-ol, benzothiazole, citronellol (Zhao et al., 2011), paenibacillin (Huang and Yousef, 2015), di-n-butyl phthalate (Deng et al., 2011), lipopeptide (Mageshwaran et al., 2012), and phenazine-1-carboxylic acid (Tupinamba et al., 2008), and systemic resistance inducers, including 2,3-butanediol, methanethiol and isoprene (Gao et al., 2010; Lee et al., 2012).

*Paenibacillus polymyxa* strains are used as soil inoculants in agriculture and horticulture as efficient plant growth promoting rhizobacteria (PGPR). Currently, comparative genomics is recognized as an important tool for identifying and understanding major biocontrol mechanisms and key functional genes among related organisms (Helfrich et al., 2014). The key genes responsible for the production of antimicrobial agents and volatile organic compounds, indoleacetic acid (IAA) synthesis, siderophore secretion, phosphate transporter, and phosphonate cluster biosynthesis in *P. polymyxa* strains, including *P. polymyxa* E681 (Kim et al., 2010), *P. polymyxa* SC2 (Ma et al., 2011), *P. polymyxa* M1 (Niu et al., 2011), *P. polymyxa* SQR-21 (Li et al., 2014), *P. polymyxa* CR1 (Eastman et al., 2014), *P. polymyxa* Sb3-1 (Rybakova et al., 2015), *P. polymyxa* YC0136 (Liu et al., 2017a), *P. polymyxa* YC0573 (Liu et al., 2017b), and *P. polymyxa* HY96-2 (Luo et al., 2018) were identified by genome sequencing and confirmed by a combination of physiological experiments.

In this study, we demonstrate the sequences and annotations of two strains *P. polymyxa* (ZF129 and ZF197), and compare the genomes with the thirteen representative *P. polymyxa* strains that are beneficial to plant growth. Strains ZF129 and ZF197 both exhibit significant broad inhibitory spectra against various plant-pathogenic fungi and bacteria and possess excellent biocontrol characteristics and potential for the biocontrol of vegetable diseases. However, their specific biocontrol mechanisms, especially at the molecular level, are still unclear. By comparing the genomic analysis with the *P. polymyxa* strains, our aim was to better understand their biocontrol mechanisms at the molecular level. Besides, secondary metabolite biosynthesis, IAA biosynthesis, phosphate solubilization, nitrogen fixation, and systemic resistance inducer production were analyzed via genomic comparison. These data will provide important insights for the study of biocontrol mechanisms and benefit the practical application of strains ZF129 and ZF197 in the field.

## Materials and Methods

### Bacterial Isolation, Culture Conditions, Genomic DNA Extraction, and Antagonistic Assays

Strain ZF129 and ZF197 were isolated from the rhizosphere soil of potatoes grown in Guyuan and Gaomi, respectively, in China. Strains ZF129 and ZF197 were cultivated in LB (Luria broth) medium at 28°C with shaking for 36 h. The morphology of the two strains was observed by scanning electron microscopy (SEM) and transmission electron microscopy (TEM; JEOL 1230 microscope). Genomic DNA was extracted from cultured ZF129 and ZF197 cells (OD_600_ = 0.8) using a TIANamp Bacteria DNA kit [Tiangen Biotech (Beijing) Co., Ltd]. The antagonistic activities of strains ZF129 and ZF197 against pathogenic bacteria and fungi were assessed through plate bioassays, and all of the experiments were repeated three times.

### Inhibitory Activities of the Cell-Free Supernatants or Volatiles From *P. polymyxa* ZF129 and ZF197 Against *Fusarium oxysporum*

The *P. polymyxa* ZF129 and ZF197 strains were cultured in LB medium at 30°C with shaking at 180 rpm in the dark for 48 h. The cell-free culture supernatant was collected by centrifugation at 6,000 × *g* for 10 min and sequentially filtered through a 0.22 μm organic filter membrane. The filtrate was used for the antimicrobial activity test.

To measure the inhibitory activity against mycelial growth, the prepared cell-free supernatant was added to agar plates (1.5% w/v agar) containing potato dextrose agar (PDA, Merck) to achieve a final concentration of 10% (v/v). LB medium was used as the control. Then, a 6-mm mycelial plug was removed from the margin of the *F. oxysporum* colony and placed in the center of the PDA plate. The plates were incubated at 28°C for 5 days and examined for fungal growth. The inhibition activity was expressed in terms of the percentage of mycelial growth inhibition and was calculated according to the following formula: inhibition (%) = [(growth in control – growth in treatment)/growth in control] × 100.

A bioassay of volatiles from *P. polymyxa* ZF129 and ZF197 against *F. oxysporum* was performed in sealed dishes using a previously described method (Tagg and Mcgiven, 1971) with some modifications. Briefly, 300 mL of bacterial culture was spread on sterile solid LB medium supplemented with 1.5% agar. A 6-mm *F. oxysporum* mycelial plug taken from the margin of the colony was then placed in the center of a new PDA agar plate. The fungal dish was immediately inverted over the bacterial dish, and the dishes were rapidly sealed with Parafilm. The dishes were incubated at 28°C in the dark until the *F. oxysporum* mycelium in the controls extended over 3/4 of the plate. Volatiles from LB medium served as controls in place of bacterial volatiles. The diameter (mm) of the fungal colonies were measured, and inhibition activity was calculated in the same way.

### Genome Sequencing, Assembly, and Annotation

The genomes of *P. polymyxa* ZF129 and ZF197 were sequenced by Igenecode, Beijing, China^1^. Whole-genome sequencing was performed using the Pacific Biosciences (PacBio) RS II platform, and a 20-kb SMRTbell template was used for library construction. The sequences were assembled *de novo* using the HGAP v.2.3 program, which uses an overlap-layout-consensus algorithm with the parameter Genome Length of 6000000. The HGAP pipeline uses the longest reads as seeds to recruit all other reads for the construction of highly accurate preassembled reads through a directed acyclic graph-based consensus procedure, which we follow with assembly using off-the-shelf long-read assemblers (Supplementary Table S1; Chin et al., 2013). Graphical views of the genome alignments were generated using CGView (Tatusova et al., 2016). The identification and annotation of the functional genes were performed using the NCBI Prokaryotic Genome Annotation Pipeline (PGAP^2^) (Tatusova et al., 2016). Transfer RNA (tRNA) and ribosome RNA (rRNA) genes were identified using tRNAscan-SE version 2.0 and RNAmmer version 1.2, respectively (Lagesen et al., 2007; Lowe and Chan, 2016). The functions of the predicted proteins were assigned through comparisons against multiple databases, including the NR (non-redundant) protein databases^3^ (Li et al., 2002), the RAST (Rapid Annotation using Subsystem Technology) analysis platform (Aziz et al., 2008), Pfam^4^, SwissProt and the enhanced COG (clusters of orthologous groups of proteins) database^5^ (Tatusov et al., 2000). In addition, SignalP 4.0 (Bendtsen et al., 2004) and TMHMM 2.0 (Krogh et al., 2001) were used to predict putative signal peptides and transmembrane helices, respectively. PHAST was used for prophage prediction (Arndt et al., 2016), and clustered regularly interspaced short palindromic repeats (CRISPRs) were identified using CRISPR finder (Grissa et al., 2008). Besides genome sequencing and assembly, all of other bioinformatics analysis were also performed by Igenecode, Beijing, China.

### Phylogenetic Analysis and Genome Comparisons

The taxonomic positions of strains ZF129 and ZF197 were determined by multilocus gene sequence analysis (MLSA) based on six housekeeping genes (16S rRNA, *gapA*, *gyrA*, *atpD*, *rpoA*, and *rho*). The gene sequences were aligned using MUSCLE and trimmed to remove ambiguously aligned regions. Subsequently, the phylogenetic tree was constructed using the maximum likelihood method in MEGA 6.0 (Tamura et al., 2013). Other available gene sequences of the *Paenibacillus* and *Bacillus* strains to be used for phylogenetic tree construction were downloaded from the NCBI database (Supplementary Table S2). According to the phylogenetic analysis, two closely related *P. polymyxa* strains with released complete genome sequences, *P. polymyxa* HY96-2 (GenBank Accession No. CP025957.1) and *P. polymyxa* SQR-21 (GenBank Accession No. CP006872.1), were selected for genome comparison with ZF129. Likewise, *P. polymyxa* J (GenBank Accession No. CP015423.1) was selected for genome comparison with ZF197. Average nucleotide identities (ANIs) (Michael and Ramon, 2009) and *in silico* DNA-DNA hybridization (DDH) (Meierkolthoff et al., 2013) were calculated using the OrthoANIu algorithm^6^ and the Genome-to-Genome Distance Calculator (GGDC)^7^, respectively. Furthermore, complete genome comparisons were conducted with the progressive alignment option of Mauve 2.3.1 comparison software using the ZF129 and ZF197 genomes as the reference genomes (Darling et al., 2004). The Mauve results were also used to create a gene-by-gene orthologous comparison, which was used to create the Veen diagrams using the R package (Venn Diagram) (Chen and Boutros, 2011).

### Analyses of Secondary Metabolite Gene Clusters

Secondary metabolite gene clusters prediction was performed using antiSMASH 2.0 on the authors’ Web servers using the default parameters^8^. Comparative analyses of secondary metabolite gene clusters identified among *P. polymyxa* ZF129, *P. polymyxa* ZF197, *P. polymyxa* HY96-2, *P. polymyxa* SQR-21, *P. polymyxa* SC2, and *P. polymyxa* J were performed based on the Kyoto Encyclopedia of Genes and Genomes database (KEGG^9^) and the GenBank database.

### Genome Mining for Genes Encoding Plant-Beneficial Traits

Functional genes involved in plant growth promotion or plant-bacterial interactions, such as those genes responsible for Indole-3-Acetic Acid (IAA) production, phosphate solubilization, and nitrogen fixation, were searched in the NCBI and KEGG databases. The identities of different functional genes at the amino acid level were compared between *P. polymyxa* ZF129, *P. polymyxa* ZF197, *P. polymyxa* HY96-2, *P. polymyxa* SQR-21, *P. polymyxa* SC2, and *P. polymyxa* J using the BLAST (Basic Local Alignment Search Tool, see text footnote 3).

## Results

### Antagonistic Characteristics of the Two Biocontrol Bacteria

**FIGURE 1:**
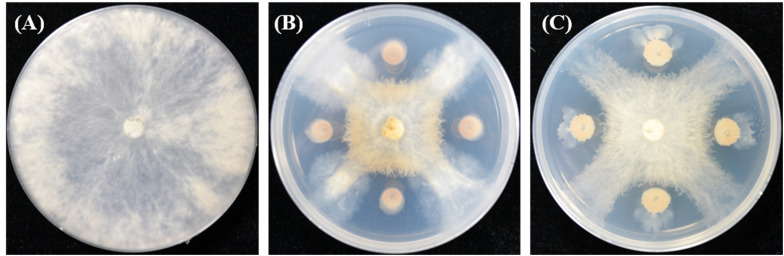
The inhibitory effect of strains ZF129 and ZF197 against *F. oxysporum* on PDA plates. **(A)** Control, **(B)** ZF129 against *F. oxysporum*, and **(C)** ZF197 against *F. oxysporum*.

To screen potential biocontrol agents for use against *F. oxysporum*, 27 bacteria were isolated from the rhizosphere of potato plants. Among these bacteria, two strains, ZF129 and ZF197, exhibited the highest inhibitory rates of 60.26 and 59.83%, respectively (Supplementary Table S3 and Figure 1). Antagonistic spectrum assays showed that strains ZF129 and ZF197 presented broad, strong antipathogenic activities against various plant-pathogenic fungi and bacteria, including *Verticillium dahlia*, *Corynespora cassiicola*, *Botrytis cinereal*, *Fusarium oxysporum*, *Colletotrichum* spp., *Rhizoctonia solani*, *Xanthomonas campestris* pv. *campestris*, *Clavibacter michiganensis* subsp. *sepedonicum*, *Ralstonia solanacearum*, *Pseudomonas syringae* pv. *tomato*, and *P. syringae* pv. *lachrymans* (Supplementary Table S4 and Supplementary Figure S2).

### Cell-Free Supernatants and Volatiles Show Antifungal Activities Against *F. oxysporum*

Strains ZF129 and ZF197 produced antifungal volatile compounds (VOCs) and inhibited the mycelial growth of *F. oxysporum* with inhibition rates of 21.82 and 26.12%, respectively (Supplementary Figure S3). The cell-free supernatant of strain ZF197 displayed a significant inhibitory effect on the growth of *F. oxysporum* (56.72%), while the inhibition rate of the cell-free supernatant of ZF129 against *F. oxysporum* was just 7.73% (Supplementary Table S5). After the antifungal bioassay, mycelial morphology was observed under an optical microscope. The mycelia treated with ZF129 or ZF197 VOCs and the cell-free supernatant of ZF197 exhibited morphological aberrations such as enlargement, distortion and shriveling, whereas no similar changes were noted in the control mycelia.

### Organism Information

*Paenibacillus polymyxa* ZF129 and ZF197 were determined to be motile, Gram-positive, rod-shaped, endospore-forming, facultative anaerobic bacteria belonging to the *Paenibacillaceae* family (Supplementary Figures S4B,D,F,H). Strains ZF129 and ZF197 grew readily on LB plates at 30°C and produced creamy white or light-yellow sticky colonies with irregular margins after 24 h of incubation (Supplementary Figures S4A,E). The interior of cell sections of ZF129 or ZF197 presented an oval or irregular shape, respectively (Supplementary Figures S4C,G).

### General Genomic Features of *P. polymyxa* ZF129 and ZF197

The complete genome of *P. polymyxa* ZF129 comprises a circular 5,703,931 bp chromosome with two additional plasmids, pAP1 and pAP2. The genome of *P. polymyxa* ZF197 is composed of a circular chromosome of 5,507,169 bp with one plasmid (Supplementary Table S6). The average G + C contents of the ZF129 and ZF197 genomes are 45.34 and 45.60%, respectively, which are similar to those of *P. polymyxa* HY96-2 (46.50%), *P. polymyxa* SQR-21 (46.50%), and *P. polymyxa* J (45.70%), but higher than those of *P. polymyxa* M1 (44.80%) and *P. polymyxa* SC2 (44.58%) (Table 1). Graphical circular genomic maps showing the genome structure and functions of *P. polymyxa* ZF129 and ZF197 are presented in Supplementary Figure S1. In total, 5,149 open reading frames (ORFs) are predicted in the genome of ZF129. In addition to 4,861 protein-coding genes (CDSs), the chromosome contains 156 RNA genes, including 110 tRNA genes, 42 rRNA operons, 4 ncRNAs and 132 pseudogenes (Supplementary Table S6). For the ZF197 strain, 5,054 ORFs are predicted in the genome. In addition to 4,902 CDSs, the chromosome possesses 109 tRNA genes, 39 rRNA genes, 4 sRNA genes, and 244 pseudogenes (Supplementary Table S6). These annotated genes are transcribed in both the positive and negative directions in terms of the direction of DNA replication (Supplementary Figure S1).

**TABLE 1:** Genomic features of *P. polymyxa* ZF129, ZF197 and other *P. polymyxa* strains.

Features	*P. polymyxa* ZF129	*P. polymyxa* ZF197	*P. polymyxa* HY96-2	*P. polymyxa* SQR-21	*P. polymyxa* M1	*P. polymyxa* SC2	*P. polymyxa* J
Size (Mb)	5.82	5.54	5.75	5.83	6.23	6.24	5.76
G + C content (%)	45.34	45.60	45.60	45.60	44.80	44.58	45.70
Replicons	One chromosome Two plasmids	One chromosome One plasmid	One chromosome	One chromosome	One chromosome One plasmid	One chromosome One plasmid	One chromosome
Total genes	5,149	5,054	4,990	5,110	5,686	5,817	5,145
Predicted no. of CDSs	4,993	4,902	4,834	4,956	5,530	5,613	4,993
Ribosomal RNAs	42	39	42	39	42	40	42
Transfer RNAs	110	109	110	111	110	160	106
Other RNAs	4	4	4	4	4	4	4
Pseudogenes	132	244	181	155	195	185	173
GenBank sequence	CP040829.1	CP042272.1	CP025957.1	CP006872.1	HE577054.1	CP002213.2	CP015423.1

The functional categorization of the CDSs of the ZF129 and ZF197 genomes was analyzed using the Clusters of Orthologous Groups of proteins (COG) database (Supplementary Figure S5). The results showed that 3,740 CDSs of ZF129 and 3,717 of the predicted genes of ZF197 were assigned to COG categories, including general function prediction only, transcription, carbohydrate transport and metabolism, amino acid transport and metabolism, signal transduction mechanisms, and ribosomal structure and biogenesis.

### Comparison of the *P. polymyxa* ZF129 and ZF197 Genomes With Other Completely Sequenced *P. polymyxa* Strains

**FIGURE 2:**
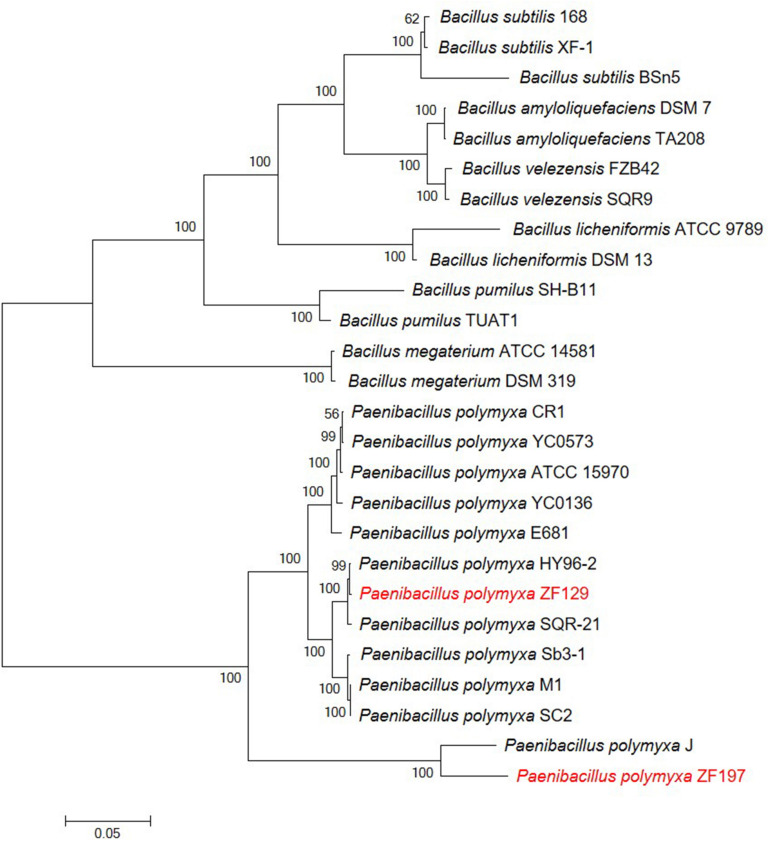
Phylogenetic tree highlighting the relative positions of *P. polymyxa* ZF129 and ZF197 among other *P. polymyxa* strains and *Bacillus* species. The phylogenetic tree was constructed based on six housekeeping genes (16S rRNA, *gapA*, *gyrA*, *atpD*, *rpoA*, and *rho*) according to the aligned gene sequences using maximum likelihoods derived from MEGA 6.0 software. Bootstrap values (1,000 replicates) are shown at the branch points. The scale bar indicates 0.05 nucleotide substitutions per nucleotide position. The GenBank accession numbers associated with the housekeeping loci of all strains can be found in Supplementary Table S2.

For the comparative genomic analysis of *P. polymyxa* ZF129 and ZF197, five publicly available complete genome sequences of *P. polymyxa* strains, including HY96-2, SQR-21, SC2, M1, and J, were selected (Table 1). To understand the relationships of *P. polymyxa* ZF129 and ZF197 with other *P. polymyxa* and *Bacillus* strains, a phylogenetic tree was constructed based on the 16S rRNA gene and five housekeeping genes (*gapA*, *gyrA*, *atpD*, *rpoA*, and *rho*) (Figure 2 and Supplementary Table S2). As expected, two primary monophyletic clades were corroborated by bootstrap values, which consisted of *P. polymyxa* and other *Bacillus* species. Strains ZF129 and ZF197 were clearly classified as *P. polymyxa* (Figure 2). Based on the observed distance relationships, *P. polymyxa* ZF129 was closely related to *P. polymyxa* HY96-2 and SQR-21. However, *P. polymyxa* ZF197 was closely related to *P. polymyxa* J. In addition, the taxonomic position of *P. polymyxa* SC2 and M1 was closer to strain ZF129 than strain ZF197.

Average nucleotide identity and DNA-DNA hybridization analyses are widely used to calculate whole-genome sequence similarities by comparing genomic data, and strains exhibiting ANI values ≥96% and DDH values ≥70% are typically regarded as the same species (Zhang et al., 2016). In this study, the ANI and DDH values between strains ZF129 and HY96-2 were 98.44 and 93.40%, respectively. Similarly, the ANI and DDH values between strains ZF197 and SC2 were 92.97 and 71.10%, respectively. Notably, the ANI and DDH values between strains ZF129 and ZF197 were 92.49 and 71.90%, respectively (Table 2). Lower ANI and DDH values were obtained when SQR-21, SC2, J, and the other three *Bacillus* standard strains were used as reference genomes. These findings revealed that strains ZF129, HY96-2, and SQR-21 clustered closely with each other and occupied the same taxonomic position.

**TABLE 2:** Percentage of average nucleotide identities (ANI)^*a*^ and *in silico* DNA-DNA hybridization (DDH)^*b*^ among the selected *Paenibacillus polymyxa* or *Bacillus* genomes.

	*Paenibacillus polymyxa* ZF129	*Paenibacillus polymyxa* ZF197	*Paenibacillus polymyxa* HY96-2	*Paenibacillus polymyxa* SQR-21	*Paenibacillus polymyxa* SC2	*Paenibacillus polymyxa* J	*Bacillus velezensis* FZB42^*T*^	*Bacillus amyloliquefaciens* DSM 7^*T*^	*Bacillus subtilis* 168^*T*^
*Paenibacillus_ polymyxa* ZF129		**92.49^*a*^**	**98.44**	**98.42**	**94.43**	**89.90**	**66.11**	**65.94**	**66.43**
		71.90^*b*^	93.40	90.60	74.30	71.50	12.70	12.70	12.70
*Paenibacillus_ polymyxa* ZF197	**92.49**		**92.36**	**92.34**	**92.97**	**89.07**	**66.19**	**66.27**	**66.73**
	71.90		74.10	71.70	71.10	68.30	12.70	12.70	12.70
*Paenibacillus_ polymyxa* HY96-2	**98.44**	**92.36**		**98.81**	**94.64**	**89.88**	**66.13**	**65.60**	**66.38**
	93.40	74.10		95.10	75.30	74.70	12.70	12.70	12.70
*Paenibacillus_ polymyxa* SQR-21	**98.42**	**92.34**	**98.81**		**94.70**	**89.92**	**66.15**	**65.91**	**66.17**
	90.60	71.70	95.10		73.80	73.20	12.70	12.70	12.70
*Paenibacillus_ polymyxa* SC2	**94.43**	**92.97**	**94.64**	**94.70**		**89.99**	**66.52**	**66.09**	**66.73**
	74.30	71.10	75.30	73.80		67.20	12.70	12.70	12.70
*Paenibacillus polymyxa* J	**89.90**	**89.07**	**89.88**	**89.92**	**89.99**		**66.34**	**66.18**	**67.08**
	71.50	68.30	74.70	73.20	67.20		12.80	12.70	12.80
*Bacillus velezensis* FZB42^T^	**66.11**	**66.19**	**66.13**	**66.15**	**66.52**	**66.34**		**94.20**	**77.12**
	12.70	12.70	12.70	12.70	12.70	12.80		80.70	33.40
*Bacillus amyloliquefaciens* DSM 7^T^	**65.94**	**66.27**	**65.60**	**65.81**	**66.09**	**66.18**	**94.20**		**77.10**
	12.70	12.70	12.70	12.70	12.70	12.70	80.70		31.30
*Bacillus subtilis* 168^T^	**66.43**	**66.73**	**66.38**	**66.17**	**66.73**	**67.08**	**77.12**	**77.10**	
	12.70	12.70	12.70	12.70	12.70	12.80	33.40	31.30	

*^*a*^ANI values were computed for pairwise genome comparison with using the OrthoANIu algorithm. The percentage of ANI was shown on the top and bolded. ^*b*^*In silico* DNA-DNA hybridization was calculated by using Genome-to-Genome Distance Calculator (GGDC). The percentage of DDH was shown on the bottom.*

**FIGURE 3:**
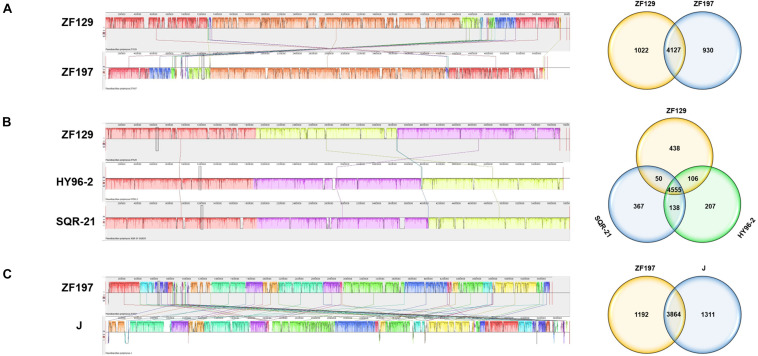
Global alignment of the genome sequences of two completely sequenced *P. polymyxa* strains against three other *P. polymyxa* genome sequences. **(A)** Mauve progressive alignment of the ZF129 and ZF197 genomes. **(B)** Mauve progressive alignment of the ZF129, HY96-2, and SQR-21 genomes. **(C)** Mauve progressive alignment of the ZF197 and J genomes. The ZF129 genome and the ZF197 genome were used as the reference genomes. Boxes with the same color indicate syntenic regions. Boxes below the horizontal strain line indicate inverted regions. Rearrangements are indicated with colored lines. The scale is in nucleotides. The Venn diagram shows the numbers of shared and unique Clusters of Orthologous Genes at the subspecies and species levels.

To evaluate the evolutionary distance among these sequenced strains in relation to several *P. polymyxa* strains, the whole-genome sequences were compared using Mauve. The alignments between ZF129 and ZF197 showed that several gene inversions or deletions were detectable in *P. polymyxa* ZF129 (Figure 3A). Compared to HY96-2 and SQR-21, a number of gene insertions or deletions and large local collinear block (LCB) inversions were also detected in *P. polymyxa* ZF129. In comparison to SQR-21, there was no significant deletion of large regions or large LCB inversions in *P. polymyxa* HY96-2 (Figure 3B). For strain ZF197, the genome sequence was aligned to *P. polymyxa* J. The results showed that regions with low similarity between the genomes occurred frequently and were distributed randomly between the ZF197 and J strains (Figure 3C). The results of the colinearity analysis were consistent with the synteny plot of the pairwise alignments from all of the above analyses.

To identify the specific genes of *P. polymyxa* ZF129 and ZF197, we compared their genome sequences with the released complete genome sequences of three *P. polymyxa* strains, HY96-2, SQR-21, and J (Figure 3). There were 4,127 conserved genes shared between strains ZF129 and ZF197. A total of 1,022 unique genes were present in the genome of ZF129, and 930 unique genes were present in the genome of ZF197. The core genome among ZF129, HY96-2 and SQR-21 was composed of 4,555 orthologous genes. ZF129 shared 106 genes with HY96-2 and 50 genes with SQR-21. Furthermore, 438 unique genes were present in the genome of ZF129, and the functions of most of these genes are still unknown. The analysis also revealed that a core genome consisting of 3,864 genes was common to strains ZF197 and J, while *P. polymyxa* ZF197 exhibited 1,192 unique genes.

### Comparison of Genes or Gene Clusters Involved in Antibiotic Synthesis

For strain ZF129, 14 gene clusters involved in secondary metabolite production were retrieved, including four clusters encoding NRPSs (non-ribosomal peptide synthetases), three encoding transAT-PKSs (trans-acyl transferase polyketide synthetases) or NRPSs, one encoding an NRPS-like protein, three encoding lanthipeptides, and one each encoding one lassopeptide, one betalactone and one bacteriocin. Ten gene clusters related to secondary metabolite production were retrieved in strain ZF197, including five encoding NRPSs and one each encoding an NRPS-like protein, one lassopeptide, one bacteriocin, one NRPS or beatlactone and one phosphonate (Table 3).

**TABLE 3:** Comparison of gene clusters and core genes involved in antibiotic biosynthesis in strains ZF129 and ZF197 as well as strains HY96-2, SQR-21, SC2, and J.

Antibiotic name	Type	Bioactive spectrum	Core gene clusters	ZF129	ZF197	HY96-2	SQR-21	SC2	J
Fusaricidin	NRPS	Fungal, G^+^bacteria	*fusA, fecS*	FGY93_ RS07740-FGY93_ RS07940	FQU75_ RS17055-FQU75_ RS17255	C1A50_ RS00290-C1A50_ RS00500	PPSQR21_ RS00295-PPSQR21_ RS00495	PPSC2_ RS29015- PPSC2_ RS29230	AOU00_ RS13080-AOU00_ RS13295
Paenilarvins	TransATPKS-NRPS	Fungal	*nrsABC*	FGY93_ RS03150- FGY93_ RS03340	NA	C1A50_ RS04650- C1A50_ RS04845	PPSQR21_ RS04670-PPSQR21_ RS04860	PPSC2_ RS33500- PPSC2_ RS33695	NA
Polymyxin	NRPS	Bacteria	*pmxABE*	FGY93_ RS11650-FGY93_ RS11810	NA	C1A50_ RS21330- C1A50_ RS21525	PPSQR21_ RS21565-PPSQR21_ RS21745	PPSC2_ RS50695- PPSC2_ RS50860	AOU00_ RS09095-AOU00_ RS09245
Tridecaptin	NRPS	G^–^bacteria	*fusAA, pmxE*	FGY93_ RS21870-FGY93_ RS22055	FQU75_ RS02970-FQU75_ RS03155	C1A50_ RS10840- C1A50_ RS11035	PPSQR21_ RS10975-PPSQR21_ RS11160	PPSC2_ RS40010- PPSC2_ RS40205	AOU00_ RS24020-AOU00_ RS24185
Bacitracin	NRPS	G^+^bacteria	*nrpS1, nrpS2, leuA5*	NA	FQU75_ RS04550-FQU75_ RS04745	C1A50_ RS24385	NA	PPSC2_ RS53660	AOU00_ RS00290- AOU00_ RS00485
Kalimantacin/Aurantinins B-D	TransATPKS-Otherks-NRPS	G^+^bacteria	*pksDEFJLMR*	FGY93_ RS17410- FGY93_ RS17670	NA	C1A50_ RS15500- C1A50_ RS15755	PPSQR21_ RS15345- PPSQR21_ RS15595	PPSC2_ RS44500- PPSC2_ RS44755	AOU00_ RS03355- AOU00_ RS03630
Paenilan	Lantipeptide	G^+^bacteria	*spaC1, spaC2, bsaB*	FGY93_ RS00440- FGY93_ RS00545	NA	C1A50_ RS07485- C1A50_ RS07590	PPSQR21_ RS07695- PPSQR21_ RS07800	PPSC2_ RS36150- PPSC2_ RS36265	NA
Paeninodin	Lassopeptide	Fungal, bacteria, virus	*asnB*	FGY93_ RS01940- FGY93_ RS02055	FQU75_ RS22660- FQU75_ RS22780	C1A50_ RS05950- C1A50_ RS06060	PPSQR21_ RS05935- PPSQR21_ RS06045	PPSC2_ RS34680- PPSC2_ RS34790	AOU00_ RS18815- AOU00_ RS18925
Paenibacillin	lanthipeptide	Fungal, bacteria	*subBC*	FGY93_ RS03415- FGY93_ RS03515	NA	C1A50_ RS22090- C1A50_ RS22155	PPSQR21_ RS22300- PPSQR21_ RS22365	NA	AOU00_ RS09800- AOU00_ RS09865
PaenicidinA/B	lanthipeptide	Fungal, bacteria	*paeBC*	FGY93_ RS09575- FGY93_ RS09670	NA	C1A50_ RS23520- C1A50_ RS23615	PPSQR21_ RS23745- PPSQR21_ RS23840	NA	AOU00_ RS11260- AOU00_ RS11350
Anabaenopeptin/nostamide	betalactone	bacteria	*nrpS1, leuA*	FGY93_ RS20370- FGY93_ RS20515	NA	C1A50_ RS12640- C1A50_ RS12785	PPSQR21_ RS12570- PPSQR21_ RS12715	NA	NA
Paenibacterin	NRPS, T1PKS	Bacteria	*grsB, tycC, pmxA, ituB, fusA*	FGY93_ RS20840- FGY93_ RS21030	FQU75_ RS05080- FQU75_ RS05250	C1A50_ RS11960- C1A50_ RS12090	PPSQR21_ RS12105- PPSQR21_ RS12250	NA	NA
Surfactin	NRPS	Bacteria, virus	*srfAA, nrsBC*	NA	FQU75_ RS15390- FQU75_ RS15605	NA	NA	NA	NA
Tauramamide	phosphonate	Bacteria	*aepX*	NA	FQU75_ RS15895- FQU75_ RS16115	NA	NA	NA	NA
Unknown	NRPS	NA	*fusAA*	NA	FQU75_ RS03885- FQU75_ RS04030	C1A50_ RS11755- C1A50_ RS11925	NA	NA	AOU00_ RS24935- AOU00_ RS25105
Unknown	NRPS, transAT-PKS	NA		FGY93_ RS02225- FGY93_ RS02475	NA	C1A50_ RS05530- C1A50_ RS05785	PPSQR21_ RS05515- PPSQR21_ RS05770	NA	NA
Unknown	Bacteriocin	NA	*kinB1*	FGY93_ RS02640- FGY93_ RS02695	FQU75_ RS22060- FQU75_ RS22115	C1A50_ RS05290- C1A50_ RS05345	PPSQR21_ RS05290- PPSQR21_ RS05345	PPSC2_ RS34090- PPSC2_ RS34140	AOU00_ RS18205- AOU00_ RS18260
Unknown	NRPS-like	NA	*lgrD*	FGY93_ RS23885- FGY93_ RS24065	FQU75_ RS01080- FQU75_ RS01265	C1A50_ RS08985- C1A50_ RS09165	PPSQR21_ RS09155- PPSQR21_ RS09335	PPSC2_ RS38125- PPSC2_ RS38310	AOU00_ RS22145- AOU00_ RS22310

*NA, not available.*

**FIGURE 4:**
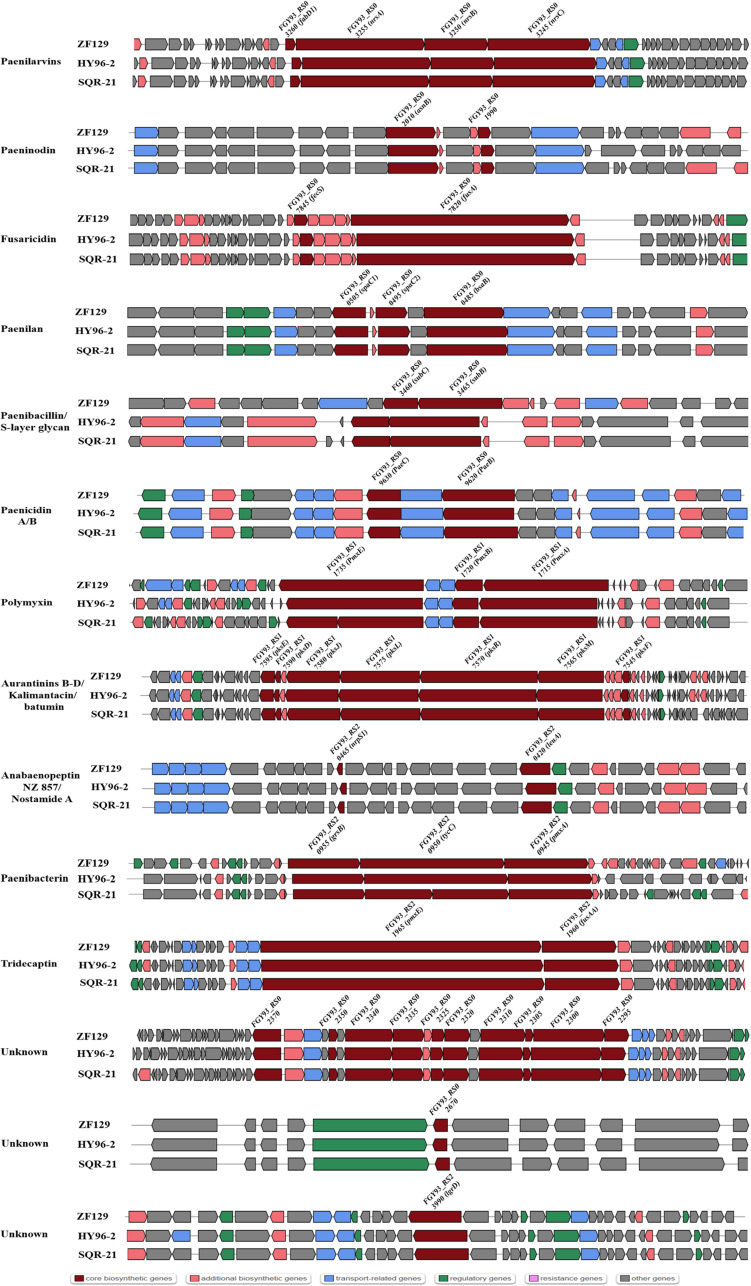
The predicted NRPS, transATPKS and peptide biosynthetic gene clusters show variation in ZF129 (top), HY96-2 (middle), and SQR-21 (bottom). Dark red indicates the core biosynthetic genes in different gene clusters among the three *P. polymyxa* strains. The core biosynthetic genes were relabeled in each gene cluster.

**FIGURE 5:**
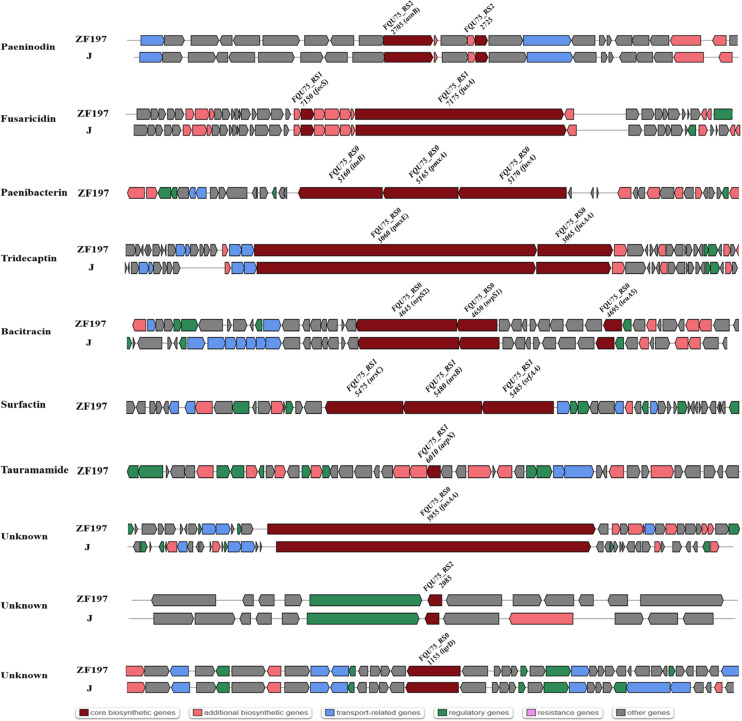
Comparisons of NRPS, transATPKS and peptide clusters in ZF197 (top) and J (bottom). Dark red indicates the core biosynthetic genes in different gene clusters among the three *Bacillus* strains. The core biosynthetic genes were relabeled in each gene cluster.

The comparison of genes or gene clusters related to antibiotic synthesis suggested that five secondary metabolites could be synthesized by strains ZF129, HY96-2, SQR-21, ZF197, and J, including fusaricidin, tridecaptin, paeninodin, and two unknown antibiotics. Furthermore, 14 gene clusters related to the biosynthesis of secondary metabolites existed in strains ZF129, HY96-2, and SQR-21 with high identities (Figure 4), and seven gene clusters associated with the biosynthesis of secondary metabolites existed in both strains ZF197 and J with high identities (Figure 5). However, three gene clusters related to the biosynthesis of secondary metabolites, including paenibacterin, surfactin and tauramamide, that were found in strain ZF197 did not exist in strains ZF129, HY96-2, SQR-21 and J. In addition, no gene clusters for the biosynthesis of paenilaryins, polymuxin, kalimantacin, paenilan, paenibacillin, paenicidin, and anabaenopeptin were detected in the ZF197 genome (Figure 5).

### Comparison of Genes Involved in Plant Growth-Promoting Traits

The key genes involved in plant growth promotion in *P. polymyxa* ZF129 and ZF197 were retrieved from the NCBI and KEGG databases, and the identities of these genes among ZF129, ZF197, HY96-2, SQR-21, and J were compared. The results indicated that six key genes (*trpC*, *trpS*, *trpE*, *trpD*, *trpB*, and *mtrB*) responsible for IAA (indole-3-acetic acid production) production were all found in the genomes of ZF129, ZF197, HY96-2, SQR-21 and J, with sequence identities exceeding 94% between *P. polymyxa* ZF129 and the other strains (Table 4). The results revealed that these strains may be expected to possess similar capacities for plant growth promotion.

**TABLE 4:** Comparison of genes involved in plant growth promotion in strains ZF129 and ZF197 as well as strains HY96-2, SQR-21, SC2, and J (sequence similarity is expressed as a percentage of the amino acid identity).

Trait	Gene name	ZF129	ZF197		HY96-2		SQR-21		SC2		J	
		Protein id	Protein id	Identity (%)	Protein id	Identity (%)	Locus tag	Identity (%)	Locus tag	Identity (%)	Locus tag	Identity (%)
Indole-3-acetic production	*trp*C	WP_126666180.1	WP_149033441.1	94.32	WP_016822202.1	98.48	WP_025365128.1	98.11	WP_013371667.1	95.45	WP_069289827.1	94.70
	*trp*S	WP_102998166.1	WP_149034229.1	96.66	WP_016819987.1	99.70	WP_025365775.1	99.39	WP_013372932.1	97.87	WP_061829859.1	98.48
	*mtr*B	WP_013371682.1	WP_063213495.1	98.68	WP_025365133.1	97.37	WP_025365133.1	97.37	WP_013371682.1	100	WP_013310730.1	97.37
	*trp*E	WP_016822204.1	WP_149033443.1	98.64	WP_016822204.1	100.00	WP_016822204.1	100.00	WP_013371669.1	99.81	WP_061829209.1	97.67
	*trp*D	WP_126666179.1	WP_149033442.1	97.70	WP_016822203.1	99.71	WP_025365129.1	99.43	WP_013371668.1	98.28	WP_069289828.1	97.41
	*trp*B	WP_031461664.1	WP_063213478.1	98.49	WP_031461664.1	100.00	WP_016822200.1	99.75	WP_013371665.1	99.25	WP_069289825.1	98.24
Phosphate solubilization	*pho*A	WP_126666660.1	WP_149035852.1	93.43	WP_045244009.1	99.08	WP_025364355.1	98.62	WP_013370018.1	97.94	WP_069289795.1	38.37
	*phn*E	WP_016820374.1	WP_149034369.1	39.35	WP_017425566.1	38.39	WP_019688655.1	38.89	WP_016324733.1	39.35	WP_028541790.1	39.35
	*phn*C	WP_113051032.1	WP_149034368.1	98.05	WP_023990372.1	99.61	WP_023990372.1	99.61	WP_025674722.1	99.22	WP_039273947.1	99.22
	*pst*B	WP_016819624.1	WP_149036033.1	98.21	WP_016819624.1	100.00	WP_016819624.1	100.00	WP_013370344.1	99.64	WP_061828683.1	98.57
	*pst*A	WP_126666903.1	WP_149036234.1	98.66	WP_016819623.1	99.66	WP_016819623.1	99.66	WP_013309592.1	99.33	WP_023987910.1	99.33
	*pst*C	WP_013370343.1	WP_063213116.1	99.03	WP_013370343.1	100.00	WP_013370343.1	100.00	WP_013370343.1	100.00	WP_013370343.1	100.00
Organic acid biosynthesis	*gnd*A	WP_013371891.1	WP_063213661.1	99.79	WP_013371891.1	100.00	WP_013371891.1	100.00	WP_013371891.1	100.00%	WP_013310934.1	99.6
	*gnd*	WP_102997927.1	WP_149032847.1	98.99	WP_025364697.1	99.33	WP_025364697.1	99.33	WP_013370842.1	99.33	WP_053325295.1	98.32
	*gnt*K	WP_019685952.1	WP_149034824.1	64.50	WP_019685952.1	100.00	WP_025363846.1	99.42	WP_013373829.1	64.89	WP_069290720.1	64.69
	*eda*	WP_013373614.1	WP_149034664.1	99.07	WP_013373614.1	100.00	WP_013373614.1	100.00	WP_013373614.1	100.00	WP_025683172.1	98.60
	*ppc*	WP_039275207.1	WP_149034421.1	100.00	WP_045245419.1	96.13	WP_025365926.1	96.24	WP_013373240.1	96.24	WP_061832059.1	99.89
	*cit*Z	WP_016819628.1	WP_149036038.1	99.19	WP_016819628.1	100.00	WP_016819628.1	100.00	WP_013370349.1	99.19	WP_069291601.1	98.11
	*glt*B	WP_126666110.1	WP_149035687.1	98.63	WP_017426451.1	99.74	WP_025364254.1	99.80	WP_013369808.1	99.28	WP_069291323.1	98.24
	*acn*A	WP_016819329.1	WP_149035411.1	98.34	WP_016819329.1	100.00	WP_016819329.1	100.00	WP_013369423.1	99.45	WP_061831538.1	98.89
	*icd*A	WP_016819629.1	WP_016819629.1	100.00	WP_016819629.1	100.00	WP_016819629.1	100.00	WP_013370350.1	99.77	WP_069291602.1	99.07
	*suc*C	WP_031463318.1	WP_149032758.1	98.70	WP_039277123.1	99.22	WP_016820942.1	99.74	WP_013370664.1	98.45	WP_061831857.1	98.19
	*suc*D	WP_013370665.1	WP_149032759.1	99.35	WP_013370665.1	100.00	WP_013370665.1	100.00	WP_013370665.1	100.00	WP_025723280.1	99.35
	*sdh*A	WP_016820039.1	WP_063209489.1	98.97	WP_016820039.1	100.00	WP_025365750.1	99.83	WP_013372871.1	99.66	WP_061830109.1	98.80
	*sdh*B	WP_019688512.1	WP_063209487.1	98.43	WP_013372870.1	99.61	WP_013372870.1	99.61	WP_013372870.1	99.61	WP_013311795.1	96.86
	*fum*C	WP_016820785.1	WP_149033219.1	99.13	WP_016820785.1	100.00	WP_025364978.1	99.57	WP_013371371.1	99.13	WP_025722755.1	97.84
	*mdh*	WP_016819630.1	WP_063213110.1	98.08	WP_016819630.1	100.00	WP_016819630.1	100.00	WP_013370351.1	97.12	WP_061828678.1	95.21
	*ldh*	WP_019685949.1	WP_063210541.1	36.33	WP_019685949.1	100.00	WP_016818814.1	99.69	WP_013369109.1	98.43	WP_029517496.1	35.37
	*pdh*A	WP_013371457.1	WP_013371457.1	100.00	WP_016821911.1	99.72	WP_013371457.1	100.00	WP_013371457.1	100.00	WP_013310511.1	98.31
	*pox*B	WP_016821074.1	WP_149032865.1	98.26	WP_045243828.1	99.48	WP_025364706.1	99.65	WP_013370864.1	98.43	WP_069291767.1	97.39
	*ack*	WP_126666189.1	NA		WP_013371630.1	99.75	WP_013371630.1	99.75	WP_013371630.1	99.75	WP_013310675.1	98.24
	*pfl*A	WP_013371803.1	WP_149033528.1	97.98	WP_045244440.1	99.21	WP_016822311.1	99.21	WP_013371803.1	100.00	WP_013310845.1	98.02
	*pfl*B	WP_016822312.1	WP_149033529.1	99.20	WP_045244439.1	99.87	WP_016822312.1	100.00	WP_013371804.1	99.47	WP_061829055.1	97.88
Nitrogen fixation	*nif*B	NA	WP_149035591.1		NA		NA		NA		WP_069291232.1	95.05
	*nif*H	NA	WP_063212489.1		NA		NA		NA		WP_061831012.1	98.61
	*nif*D	NA	WP_149035592.1		NA		NA		NA		WP_069291233.1	97.72
	*nif*K	NA	WP_149035593.1		NA		NA		NA		WP_069291234.1	97.25
	*nif*E	NA	WP_149035594.1		NA		NA		NA		WP_069291235.1	97.35
	*nif*N	NA	WP_149035595.1		NA		NA		NA		WP_069291236.1	95.63

*NA, not available. Genes were identified using annotations provided in GenBank followed by BLASTx searches of the genomes using previously characterized homologs.*

In addition, six key genes related to phosphate solubilization were all retrieved from the five *P. polymyxa* strains (Table 4). The sequence identities of the four *pstA-C* and *phnC* genes between ZF129 and the other four strains were 98.05–100.00%, while the sequence identities of the *phnE* gene among the five strains were under 40%. In addition, high consistency of *phoA* was found between strains ZF129, ZF197, HY96-2, and SQR-21; however, the sequence identity between ZF129 and J was only 38.37%. Furthermore, 20 genes responsible for organic acid biosynthesis were all found in the genomes of ZF129, ZF197, HY96-2, SQR-21 and J, most of which were highly conserved (Table 4). The identities of the corresponding genes between ZF129 and HY96-2 and SQR-21 (96.13–100.00%) were higher than those between the ZF129, ZF197, and J strains (35.37–100.00%), especially for the *ldh* and *gntK* genes. The *ack* gene was found in strains ZF129, HY96-2, SQR-21 and J with sequence identities of 98.24–99.75% but was not detected in strain ZF197. Notably, key genes related to nitrogen fixation (*nifB, nifH, nifD, nifK, nifE*, and *nifN*) were only found in the genomes of strains ZF197 and J (Table 4), with sequence identities ranging from 95.05% to 98.61%.

### Comparison of Genes Involved in Resistance Inducer Synthesis

Key genes associated with the synthesis of resistance inducers in *P. polymyxa* were retrieved from the KEGG database, and the identities of these genes between ZF129, ZF197, HY96-2, SQR-21, and J were compared. The results indicated that key genes for 2,3-butanediol (*budA*, *ilyN*), methanethiol (*metH*, *metE*), and isoprene (*idi*, *lytB*, *gcpE*, *ispF*, and *ispE*) synthesis were found in the genomes of all these strains, with sequence identities exceeding 92% (Table 5). The identities of the corresponding genes between ZF129 and ZF197, HY96-2, and SQR-21 (94.43–100%) were higher than those between ZF129 and J (92.62–99.19%).

**TABLE 5:** Comparison of genes involved in the synthesis of resistance inducers in strains ZF129 and ZF197 as well as strains HY96-2, SQR-21, SC2, and J (sequence similarity is expressed as a percentage of the amino acid identity).

Resistance inducer	Gene	Product	ZF129		ZF197		HY96-2		SQR-21		SC2		J
			Locus tag	Locus tag	Identity (%)	Locus tag	Identity (%)	Locus tag	Identity (%)	Locus tag	Identity (%)	Locus tag	Identity (%)
2,3-Butanediol	*bud*A	Acetolactate decarboxylase	WP_016821069.1	WP_149032860.1	96.37%	WP_016821069.1	100.00%	WP_025364704.1	99.60%	WP_013370859.1	97.58%	WP_025722973.1	95.56
	*ilv*N	Acetolactate synthase small subunit	WP_016820552.1	WP_016820552.1	100.00	WP_016820552.1	100.00	WP_016820552.1	100.00	WP_013370080.1	99.38	WP_023987760.1	98.76
Methanethiol	*met*H	Methionine synthase	WP_044788161.1	WP_149033329.1	98.25	WP_045244486.1	99.74	WP_039269038.1	99.74	WP_043885986.1	99.56	WP_069289676.1	97.99
	*met*E	5-methyltetrahydro-pteroyltriglutamate- homocysteine S-methyltransferase	WP_017428578.1	WP_149034631.1	94.43	WP_039276423.1	99.35	WP_025366075.1	99.09	WP_013373554.1	96.11	WP_069290615.1	93.39
Isoprene	*idi*	Type 2 isopentenyl-diphosphate Delta-isomerase	WP_126666423.1	WP_149034550.1	94.81	WP_103042605.1	95.63	WP_025366018.1	98.63	WP_013373457.1	97.81	WP_069290552.1	92.62
	*lyt*B	4-hydroxy-3 -methylbut-2-enyl diphosphate reductase	WP_025364457.1	WP_064795308.1	97.80	WP_031463165.1	99.06	WP_025364457.1	100	WP_014599573.1	98.43	WP_013309434.1	97.80
	*gcp*E	Flavodoxin-dependent (E)-4-hydroxy-3-methylbut-2-enyl-diphosphate synthase	WP_017427410.1	WP_063212213.1	99.73	WP_017427410.1	100.00	WP_017427410.1	100.00	WP_013372694.1	99.73	WP_025718670.1	99.19
	*isp*F	2-C-methyl-D-erythritol 2,4-cyclodiphosp-hate synthase	WP_016818638.1	WP_063211188.1	98.10	WP_016818638.1	100.00	WP_016818638.1	100.00	WP_013373286.1	98.10	WP_025723806.1	96.84
	*isp*E	4-(cytidine 5’-diphospho)-2-C-methyl-D-erythritol kinase	WP_025676573.1	WP_149034923.1	99.30	WP_025676573.1	100.00	WP_016818920.1	99.65	WP_013368672.1	99.65	WP_069290753.1	98.94
													

## Discussion

Bacteria of *P. polymyxa* are agriculturally important microbes and are widely studied for their plant growth-promoting abilities. After more than 100 years of study and analysis of the *Paenibacillus* genus, it was reclassified into a separate family, *Paenibacilliaceae*, and was designated as the family’s type genus (Padda et al., 2017). Currently, the previous reports concerning the complete genome of *P. polymyxa* mainly focused on the general features of the genome and analysis of the effect of this species on promoting growth, but rarely involved the analysis of the biocontrol mechanism (Luo et al., 2018). *P. polymyxa* ZF129 and ZF197 were isolated from the rhizosphere of potato plants to obtain potential biocontrol agents for use against *F. oxysporum*. They displayed broad antipathogenic activities and potential for growth promotion. However, their specific biocontrol mechanisms, especially at the molecular level, are still unclear. In this study, the complete genomes of *P. polymyxa* ZF129 and ZF197 were sequenced and compared with those of other *P. polymyxa* strains to better understand their biocontrol mechanisms at the molecular level. Phylogenetic analysis were performed to determine the taxonomic position of ZF129 and ZF197 and their relationships with other representative *P. polymyxa* strains. To clarify the differences in the biocontrol mechanisms between strains ZF129 and ZF197 and other *P. polymyxa* strains, various genes involved in secondary metabolite biosynthesis, IAA biosynthesis, phosphate solubilization, nitrogen fixation, and systemic resistance inducer production were analyzed via genomic comparison. The results of genome comparison revealed that the genome of strain ZF129 and ZF197 exhibits some degree of variation. And this finding possibly provides an in-depth understanding of the genome architecture of *P. polymyxa*, revealing great potential for the application of this bacterium in the fields of agriculture and horticulture as a PGPR.

### Genome Comparisons Among *P. polymyxa* Strains

In this study, the evolutionary position of ZF129 and ZF197 relative to other eleven *P. polymyxa* strains which were sequenced previously were determined by multi-locus sequence analysis (MLSA). Phylogenetic trees, which were constructed based on the 16S rRNA gene and five housekeeping genes (*gapA, gyrA, atpD, rpoA*, and *rho*) using maximum likelihood methods, show significant difference with each other. The phylogenetic trees show that the eleven *P. polymyxa* strains (CR1, YC0573, ATCC15970, YC0136, E681, HY96-2, SQR-21, Sb3-1, M1, SC2, and ZF129) form a monophyletic group. The strain ZF129 was clustered with HY96-2 and SQR-21 in Poly-clade subspecies. However, two *P. polymyxa* strains (J and ZF197) form the other monophyletic group. Population structure analysis also support that the Poly-clade strains evolved from a common ancestor.

General features of the seven completely sequenced *P. polymyxa* genomes are presented in Table 1. Immediately evident is the variation in genome size and differences in plasmid content between ZF129 and ZF197, with two plasmids (116,622 bp) or one plasmid (32,065 bp) present in ZF129 or ZF197 (Supplementary Table S6). Genome size varies between 5.70 Mb in ZF129 to 5.51 Mb in ZF197 excluding plasmids, with chromosome coding DNA sequences (CDS) varying between 4,993 and 4,902, respectively. The mean G + C% of seven species is 45.32% and no strain deviates from the mean by >0.6%. The plasmids of SC2 and M1 have a lower G + C% in accordance with previously reported work (Eastman et al., 2014). With the inclusion of plasmids in the calculation, ZF129 has the larger genome and the more CDS (5.82 Mb, 4,993 CDS) with ZF197 in a close second in terms of size, albeit with significantly fewer CDS (5.54 Mb, 4,902 CDS). This difference of 91 CDS between ZF129 and ZF197 may be associated with their different plasmids (Table 1 and Supplementary Table S6). In addition, the remarkable difference of genome annotation and function prediction had been exhibited using different databases, including RAST, Pfam, SwissProt, COG, SignalP, TMHMM, PHAST, and CRISPR Finder (Supplementary Table S6). The differences in CDS between ZF129 and ZF197 is likely the result of different annotation methods employed, which can result in large discrepancies in the total number of genes identified in a genome (Richardson and Watson, 2013).

Average nucleotide identity and DNA-DNA hybridization were used as effective tools for phylogenetic analysis at the genomic level. Strains with ANI values >96% and DDH values ≥70% are considered to be the same species (Zhang et al., 2016). Genome relatedness of the strain ZF129 and ZF197 with *P. polymyxa* strains from different branches of the phylogenetic tree was analyzed on the basis of ANI and DDH. ANI and DDH values among the representative *P. polymyxa* strains are shown in Table 2, and according to the ANI and DDH values, the complete genome of ZF129 was most similar (ANI value of 98.44% and DDH value of 93.40%) to that of HY96-2, which is also in the same monophyletic group. However, the ANI and DDH values between ZF197 and four other *P. polymyxa* genomes were no significant difference except strain J. Lower ANI similarity (<90%) was obtained when *P. polymyxa* J were used as reference genomes and DDH values were lower than 70%. This genetic difference maybe due to host-specific strain diversity, adaptation and ecological niche. A possible explanation for the result may that strain ZF129 and ZF197 belonged to different subspecies in *P. polymyxa* subspecies.

Horizontal gene transfer plays an essential role in the diversification of lineages in bacteria, especially with respect to the evolution of defined lineage, species and subspecies (Nowell et al., 2014). A global alignment of ZF129, ZF197, HY96-2, SQR-21, and J chromosomes was performed using Mauve and visualized as local collinear blocks (LCBs) to glean global information into the nucleotide level similarity amongst the sequenced *P. polymyxa* genomes (Figure 3). The nucleotide level similarity between ZF129 and HY96-2 is markedly higher than the similarity between any other grouping of strains, demonstrating the close relationship of these two strains and supporting our phylogeny showing the ZF129 and HY96-2 strains forming a sub-clade within the *P. polymyxa* species. Also, readily noticeable is unsimilarity of the ZF197 chromosome compared to any other *P. polymyxa* strains, with many strain-specific, low similarity regions dispersed throughout the ZF197 genome. Based on the comparative analysis, horizontal gene transfer obviously emerged among the *P. polymyxa* strains, but the ZF129 genome was highly syntenic with HY96-2, which confirmed the phylogenetic relationship analysis described above (Table 2 and Figure 2).

### Antimicrobial Compound Production

Comparisons of the genes and gene clusters related to antibiotic synthesis demonstrated more significant differences between the five *P. polymyxa* strains. In terms of their gene sequences, strains ZF129 and HY96-2 and SQR-21 exhibited relatively high homology, as did strains ZF197 and J. Regarding the control of fungi, fusaricidins are the main antifungal secondary metabolites produced by *P. polymyxa*, and the corresponding gene clusters were found in all five strains. Fusaricidin is a peptide antibiotic consisting of a group of cyclic depsipeptides with an unusual 15-guanidino-3-hydroxypentadecanoic acid moiety bound to a free amino group (Kajimura and Kaneda, 1996) that has been identified as the most important antifungal compound produced by *P. polymyxa* (Kajimura and Kaneda, 1997). Many members of the fusaricidin family have been isolated from *P. polymyxa*, including fusaricidins A-D and LI-F03 to LI-F08 (Han et al., 2012; Liu et al., 2018). The fusaricidin biosynthetic gene cluster (BGC) contains 7 ORFs (*fusB*, *fusC*, *fusD*, *fusE*, *fusF*, *fusG*, and *fusTE*) totaling 32.4 kb and one large ORF (*fusA*) of approximately 23.7 kb, encoding a six-module non-ribosomal peptide synthetase involved in fusaricidin production (Li and Jensen, 2008). Indeed, fusaricidins show very high antifungal activities against many plant-pathogenic fungi, especially *F. oxysporum* (Raza et al., 2009). In addition, fusaricidins from *P. polymyxa* A21 exhibit excellent antagonistic activity against *B. cinerea* on tomato (Liu et al., 2018); fusaricidin B is particularly effective against *Candida albicans* and *Saccharomyces cerevisiae* (Kajimura and Kaneda, 1996); and the LI-F-type antibiotics LI-F03, LIF04, LI-F05, LI-F06, LI-F07, and LI-F08 produced by *P. polymyxa* L-1129 and *P. polymyxa* I exhibit excellent activities against *Staphylococcus aureus* (Kurusu et al., 1987; Kuroda et al., 2000). Furthermore, gene clusters related to the production of paenilarvins, a class of iturin-like lipopeptide secondary metabolites with broad-spectrum antifungal activities (Sood et al., 2014), were also found in strains ZF129, HY96-2 and SQR-21, but not in ZF197 and J. The different antifungal activities of strains ZF129 and ZF197 identified in this study may have been due to the variation in their gene clusters related to antifungal metabolite synthesis or the production of different antifungal compounds.

Regarding the control of bacteria, different antibacterial metabolites were predicted to be produced in the five strains using the antiSMASH database, including polymyxin, tridecaptin, bacitracin, kalimantacin, paenilan, and paenibacterin. Polymyxin, which is a kind of non-ribosomal lipopeptide antibiotic, was first isolated from *P. polymyxa* in 1947, and at least 15 unique polymyxins have been reported (Kim et al., 2010). The gene clusters for polymyxin synthesis span a 40.6 kb region and consist of five ORFs, designated *pmxA*, *pmxB*, *pmxC*, *pmxD*, and *pmxE* (Choi et al., 2009). Polymyxin possesses broad-spectrum antibacterical activities, especially against Gram-negative bacteria, and has been recognized as one of the main antibacterial metabolites synthesized by *P. polymyxa* (Xu et al., 2018). Notably, *P. polymyxa* ZF197 also exhibited excellent antibacterial activities against many plant-pathogenic bacteria, although no genes or gene clusters involved in the synthesis of polymyxin were found on its chromosome. The strong antibacterial activity of *P. polymyxa* ZF197 may be associated with other antibacterial metabolites, which will need to be confirmed in further experiments. Tridecaptin is a kind of lipopeptide compound that includes two main members, tridecaptin A and tridecaptin B (Cochrane et al., 2015a,b, 2016). All five *P. polymyxa* strains were found to contain tridecaptin-related gene clusters, although the identities of the gene clusters between strains ZF129, ZF197, HY96-2, and SQR-21 were clearly higher than those between strains ZF129 and J. In addition, paenibacillin is a recently discovered lantibiotic from *P. polymyxa* OSY-DF that shows potent activities against bacteria such as *Listeria monocytogenes* and *S. aureus* (He et al., 2008). The core gene clusters *pae A*, *paeB*, and *paeP* are reported to be closely related to the biosynthesis of paenibacillin (Huang and Yousef, 2015), but no homologous gene clusters were found in the genome of strain ZF197. Moreover, bacitracin, which belongs to a class of polymyxin antibiotics, is produced at high levels by *B. subtilis* (Johnson et al., 1945), although the relevant gene clusters were also retrieved in strains ZF197 and J. Bacitractins A, B1, B2, and B3 are regarded as the main active components with potent antimicrobial activities against Gram-positive bacteria, especially against coccus and bacillus bacteria (Suleiman et al., 2017).

It is worth noting that the antibacterial metabolites predicted to be produced by strains ZF129 and ZF197 were significantly different, suggesting that some secondary metabolites may present similar targets in the inhibition of plant pathogens. Interestingly, the study showed that the cell-free supernatant of *P. polymyxa* ZF197 displayed a more significant inhibitory effect on the growth of *F. oxysporum* than did that of *P. polymyxa* ZF129 (Supplementary Figure S3), although the ZF129 and ZF197 strains both harbored the gene cluster involved in the production of fusaricidin, which was the main metabolite responsible for resistance to *F. oxysporum*. In the antiSMASH database, three secondary metabolites, surfactin, tauramamide and bacitracin, were found in strain ZF197 but not in ZF129. The reason for this unexpected finding is uncertain, but it will be important to determine the relationship between the high efficiency of ZF197 against *F. oxysporum* and the production of special secondary metabolites. Moreover, four gene clusters encoding unknown secondary metabolites were also found on the genome of strain ZF129 or ZF197; as rare antibiotics, their functions and bioactive spectra require further confirmation. In summary, the genome features of ZF129 and ZF197 showed that they present potential for application in the field to control plant diseases.

### IAA Production

Indoleacetic acid is a primary plant hormone synthesized by plant-associated bacteria that has a profound effect on enhancing plant growth and development (Spaepen et al., 2007). *P. polymyxa* strains have been reported as effective plant growth-promoting bacteria, and one of their beneficial characteristics is the production of auxin-related phytohormones, especially IAA (Timmusk et al., 2005). Five different IAA biosynthetic pathways have been identified in bacteria according to the different intermediates produced during IAA biosynthesis by using tryptophan as a precursor (Patten and Glick, 1996), which mainly include indole-3-acetamide (IAM), indole-3-pyruvate (IPyA), indole-3-ethanol (TOL), indole-3-acetonitrile (IAN), and tryptamine (TAM) (Spaepen et al., 2007). *P. polymyxa* has been proven to synthesize IAA from the main precursor in a dependent manner and to possess more than one Trp-dependent biosynthetic pathway (Michael et al., 1997). It has been reported that the IPyA pathway is the main IAA production mechanism employed by *P. polymyxa* (Eastman et al., 2014). The *ipdC* gene, encoding a key enzyme in the IPyA pathway, has been detected in many *P. polymyxa* strains, including E681, CR1, and M1 (Phi et al., 2008). In our study, *ipdC* was not retrieved in the genomes of ZF129 and ZF197, whereas some *trp* genes were found in the genomes. The results suggested that ZF129 and ZF197 may be able to produce IAA in a tryptophan-independent manner, which needs to be conclusively demonstrated.

### Phosphate Solubilization and Assimilation

Phosphorus (P) is one of the major nutrients in plants, second only to nitrogen in terms of its requirement, and is considered a major essential macronutrient for the growth and development of plants (Passariello et al., 2006). The mineralization of most organic phosphorus compounds is carried out by phosphatases because plants can only utilize P in inorganic form. It has been reported that solubilization of mineral phosphates by bacteria is typically achieved through gluconic acid production. *gcd* (encoding glucose-1-dehydrogenase) and *gad* (encoding gluconic acid dehydrogenase) are the main genes responsible for the production of gluconic acid and its conversion (Werra et al., 2009). However, these two genes were not detected in the genomes of ZF129 and ZF197, although *gcd* and *gad* have been reported in *P. polymyxa* strains M1 and E681 (Xie et al., 2016). Another rich source of phosphate in soil is that trapped in the form of phosphonate, and the phosphonate gene cluster (*phn*) is responsible for the bacterial degradation of phosphonates, which release biologically available phosphate for nearby plants. Many *P. polymyxa* strains have been reported to carry *phn* genes (*phnABCDEWXM*), including strains CR1 and E681 (Xie et al., 2016); however, our comparative genomic analysis revealed that ZF129 and ZF197 do not carry the complete *phn* cluster, only possessing *phnC* and *phnE*. The variations in these genomes might be attributed to gene gain and loss events during evolution.

In addition, the PST (phosphate-specific transport) system, a high-affinity, low-velocity, free-Pi transport system, serves as a major Pi transport system in *B. subtilis* (Xie et al., 2016). The *pst* operon of *B. subtilis* contains *pstS*, *pstC*, *pstA*, *pstB1* and *pstB2*. PstC and PstA are two integral inner membrane proteins, and PstB of *Escherichia coli* (or PstB1 and PstB2 for *B. subtilis*) is an ATP-binding protein (Chan and Torriani, 1996). Our comparative genomic analysis revealed that the strains ZF129 and ZF197 carry the *pst* operon (*pstC*, *pstA*, and *pstB*), and this operon might be associated with phosphonate uptake in response to phosphate deficiency, which needs to be further verified.

### Nitrogen Fixation

Nitrogen is an important limiting element for plant growth and production in agricultural systems since plants only absorb reduced forms of nitrogen, such as ammonia and nitrates. Nitrogenase is an oxygen-sensitive dinitrogen reductase that is produced by certain microorganisms (Bohlool et al., 1992). It can convert inert atmospheric nitrogen (N_2_) into ammonium (NH_4_), thereby improving plant growth and crop yields by increasing the concentration of biologically available nitrogen (Oldroyd and Dixon, 2014). Nitrogen fixation is mainly catalyzed by Mo-nitrogenase. The ability to fix nitrogen is found among a limited number of archaeal and bacterial taxa with a wide distribution, including *Proteobacteria, Firmicutes*, *Cyanobacteria*, *Actinobacteria* and *Chlorobi* (Dos Santos et al., 2012). *P. polymyxa* has been reported to possess the capacity for nitrogen fixation (Heulin et al., 1994). For instance, *P. polymyxa* P2b-2R can grow on N-free medium, consistently reduces acetylene in an acetylene reduction assay, and harbors *nif* gene clusters encoding nitrogenase enzymes (Bal et al., 2012; Anand et al., 2013). However, the contents and organization of nitrogen fixation (*nif*) genes vary significantly among the different N_2_-fixing organisms, which raises the question of the origins and evolution of Monitrogenase (Hartmann and Barnum, 2010). For example, 20 *nif* genes are found within a 24-kb cluster in *Klebsiella pneumoniae* (Arnold et al., 1988), whereas in *Azotobacter vinelandii*, the *nif* genes are dispersed and distributed as two clusters in the genome (Setubal et al., 2009). It has been reported that *P. polymyxa* and *P. terrae* likely derived from the same ancestor possessing *nif* gene clusters, and at least 9 of these genes (*nifBHDKENXhesAnifV*) have been found to be related to nitrogen fixation (Xie et al., 2014). The *nifHDK* gene cluster encoding Mo-nitrogenase is responsible for fixing nitrogen, while *nifBENX* and *nifV* are responsible for the synthesis and maturation of the FeMo cofactor. The *hesA* gene encodes an NAD/FAD-binding protein involved in molybdopterin and thiamine biosynthesis (Wang et al., 2013). However, the *nif* gene cluster is not distributed in all the sequenced *P. polymyxa* strains, and not all the *nif* genes are identical (Xie et al., 2014). Our results were consistent with these findings, as a minimal *nif* cluster (*nifBHDKEN*) was identified only in *P. polymyxa* ZF197 and *P. polymyxa* J and not in three other *P. polymyxa* strains, ZF129, HY96-2 and SQR-21. Nevertheless, the 7 *nif* genes identified in ZF197 are of particular interest because of their potential use as a source of transferable genetic elements related to nitrogen fixation to facilitate the development of genetically modified PGPR.

### Induced Systemic Resistance

Induced systemic resistance is one of the biocontrol mechanisms whereby beneficial microorganisms control plant diseases (Bargabus et al., 2004). ISR, which is a different form of systemic resistance from systemic acquired resistance (SAR), responds to certain non-pathogenic rhizobacteria and effectively opposes multiple pathogens (Feys and Parker, 2000). It has been reported that biocontrol agents can generate and release systemic resistance inducers such as VOCs (e.g., 2,3-butanediol, methanethiol, isoprene, and butyl acetate) into the surrounding environment, thereby triggering the defensive mechanisms of plants (Lee et al., 2012; Shi et al., 2017). VOCs emitted by *Bacillus* spp. were shown to be novel determinants of ISR elicitation in *Arabidopsis* (Ryu et al., 2004). The VOCs released from *P. polymyxa* have also been proven to induce systemic resistance in *Arabidopsis* to control the foliar pathogen *P. syringae* pv. *maculicola* ES426 (Lee et al., 2012). In this study, all five *P. polymyxa* strains contained key genes associated with VOC (2,3-butanediol, methanethiol, and isoprene) production, although their sequence identities were different. The results indicated that the ZF129, ZF197, HY96-2, SQR-21, and J strains could induce similar systemic resistance in plants but with varying efficacies owing to the differences in the related genes.

## Data Availability Statement

The complete genome sequences of *P*. *polymyxa* ZF129 and ZF197 have been deposited in NCBI GenBank under accession numbers CP040829.1, CP040830.1, CP040831.1, CP042272.1, and CP042273.1, respectively. The two strains have also been deposited in the China General Microbiological Culture Collection Center (CGMCC) for Type Culture Collection under accession numbers 17631 and 17632, respectively.

## Author Contributions

QW and J-YL conceived and designed the experiments. J-YL performed the experiments. J-YL, T-TG, and QW analyzed the data and wrote the manuscript. All authors have read and approved the final manuscript.

## Conflict of Interest

The authors declare that the research was conducted in the absence of any commercial or financial relationships that could be construed as a potential conflict of interest.

## Footnotes

1 http://www.igenecode.com/

2 https://www.ncbi.nlm.nih.gov/genome/annotation_prok/

3 https://blast.ncbi.nlm.nih.gov/Blast.cgi

4 http://pfam.xfam.org/

5 https://www.ncbi.nlm.nih.gov/COG/

6 https://www.ezbiocloud.net/tools/orthoaniu

7 http://ggdc.dsmz.de/ggdc.php

8 https://antismash.secondarymetabolites.org

9 http://www.genome.jp/kegg/

## Supplementary Material

Supplementary Figure 1 Graphical circular maps of the *P. polymyxa* ZF129 chromosome and plasmids pAP1 and pAP2 as well as the *P. polymyxa* ZF197 chromosome and plasmid pAP197 generated with CGview Server. From the outside to the center, rings 1 and 12 show protein-coding genes oriented in the forward (colored by COG categories) and reverse (colored by COG categories) directions, respectively. Ring 13 shows the G + C% content plot (black), and the innermost ring shows GC skews, where green indicates positive values and purple indicates negative values.

Supplementary Figure 2 Antagonistic assays of *P. polymyxa* ZF129 and ZF197 against various plant pathogens. **(A,a)** *Verticillium dahlia*, **(B,b)** *Corynespora cassiicola*, **(C,c)** *Botrytis cinereal*, **(D,d)** *Fusarium oxysporum*, **(E,e)** *Colletotrichum* spp., **(F,f)** *Rhizoctonia solani*, **(G,g)** *Xanthomonas campestris* pv. *campestris*, **(H,h)** *Clavibacter michiganensis* subsp. *sepedonicum*, **(I,i)** *Ralstonia solanacearum*, **(J,j)** *Pseudomonas syringae* pv. *tomato*, **(K,k)** *Pseudomonas syringae* pv. *lachrymans*, **(L,l)** control.

Supplementary Figure 3 Inhibitory activities of the volatiles or cell-free supernatants of *P. polymyxa* ZF129 and ZF197. **(A)** LB plate. **(B)** Volatiles of ZF129. **(C)** Volatiles of ZF197. **(D)** LB medium. **(E)** Cell-free supernatant of ZF129. **(F)** Cell-free supernatant of ZF197.

Supplementary Figure 4 General characteristics of *P. polymyxa* ZF129 and ZF197. **(A,E)** Images of ZF129 and ZF197 colony morphology. **(B,F)** images of ZF129 and ZF197 cells obtained using transmission electron microscopy. **(C,G)** images of ZF129 and ZF197 cell sections obtained using transmission electron microscopy (Hitachi 7700, Japan), **(D,H)** images of ZF129 and ZF197 cells obtained using scanning electron microscopy (Hitachi SU8010, Japan).

Supplementary Figure 5 COG functional categorization of two sequenced *P. polymyxa* genomes. COG functional categorization was performed using tools available in the Clusters of Orthologous Groups of proteins database. The left panel indicates the COG functional classification of ZF129, and the right panel indicates the COG functional classification of ZF197.

Supplementary Table 1 The genome was assembled using the HGAP assembly pipeline in this study.

Supplementary Table 2 The locus tag information of 16S rRNA genes and five housekeeping genes used for phylogenetic tree construction in this study.

Supplementary Table 3 Inhibition rates of 27 biocontrol bacteria against *F. oxysporum*.

Supplementary Table 4 The effects of strains ZF129 and ZF197 against various plant pathogens.

Supplementary Table 5 Inhibitory activities of the volatiles or cell-free supernatants of *P. polymyxa* ZF129 and ZF197.

Supplementary Table 6 Genome statistics.
