# Supplementary figures and images for: Comparative and Functional Analyses of Two Sequenced Paenibacillus polymyxa Genomes Provides Insights Into Their Potential Genes Related to Plant Growth-Promoting Features and Biocontrol Mechanisms
Source: Front Genet. 2020 Dec 17;11:564939. doi: 10.3389/fgene.2020.564939 (PMC7773762; doi:10.3389/fgene.2020.564939)

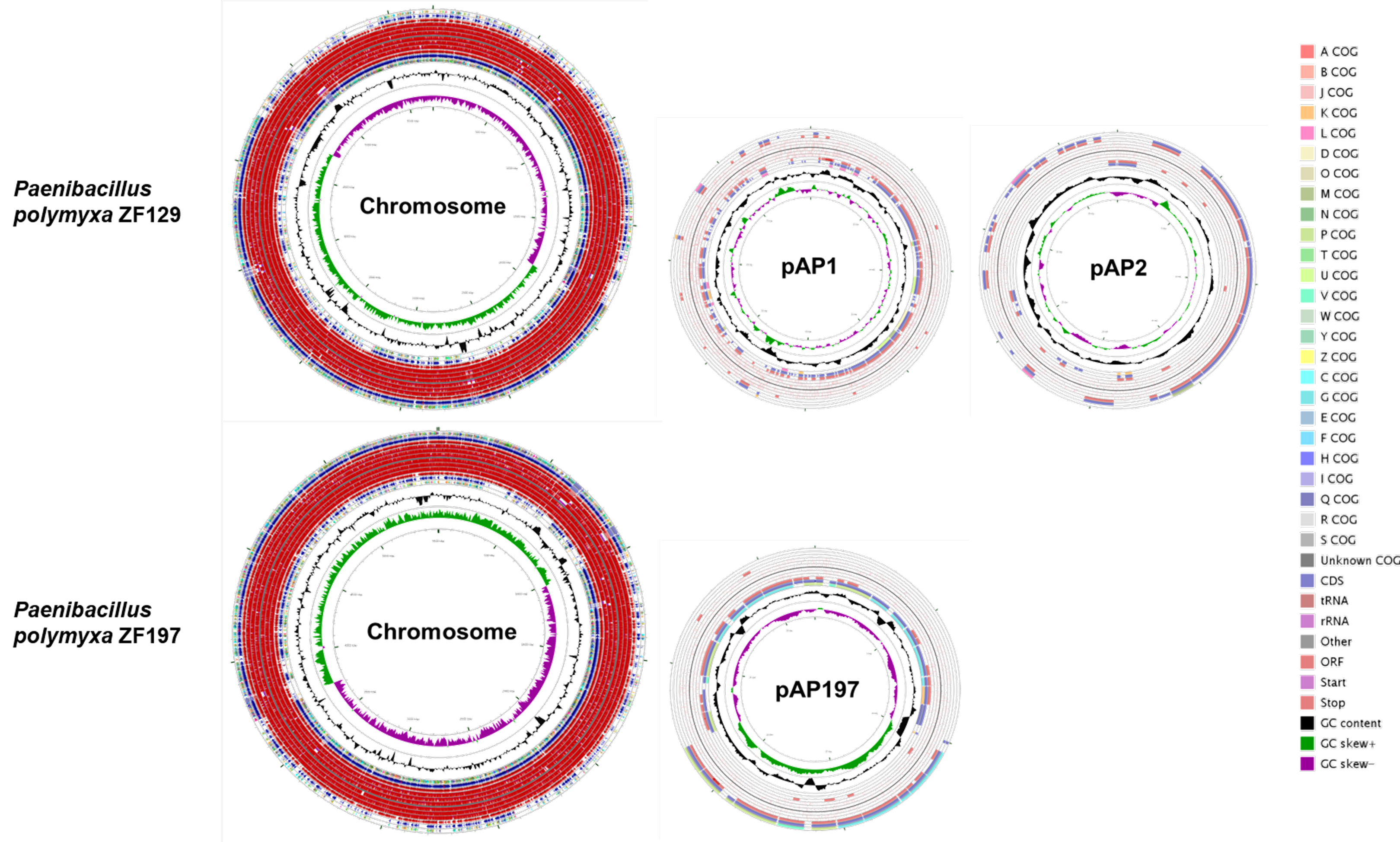

Supplement: Supplementary Figure 1 — Graphical circular maps of the P. polymyxa ZF129 chromosome and plasmids pAP1 and pAP2 as well as the P. polymyxa ZF197 chromosome and plasmid pAP197 generated with CGview Server. From the outside to the center, rings 1 and 12 show protein-coding genes oriented in the forward (colored by COG categories) and reverse (colored by COG categories) directions, respectively. Ring 13 shows the G + C% content plot (black), and the innermost ring shows GC skews, where green indicates positive values and purple indicates negative values. [file Image_1.TIF]

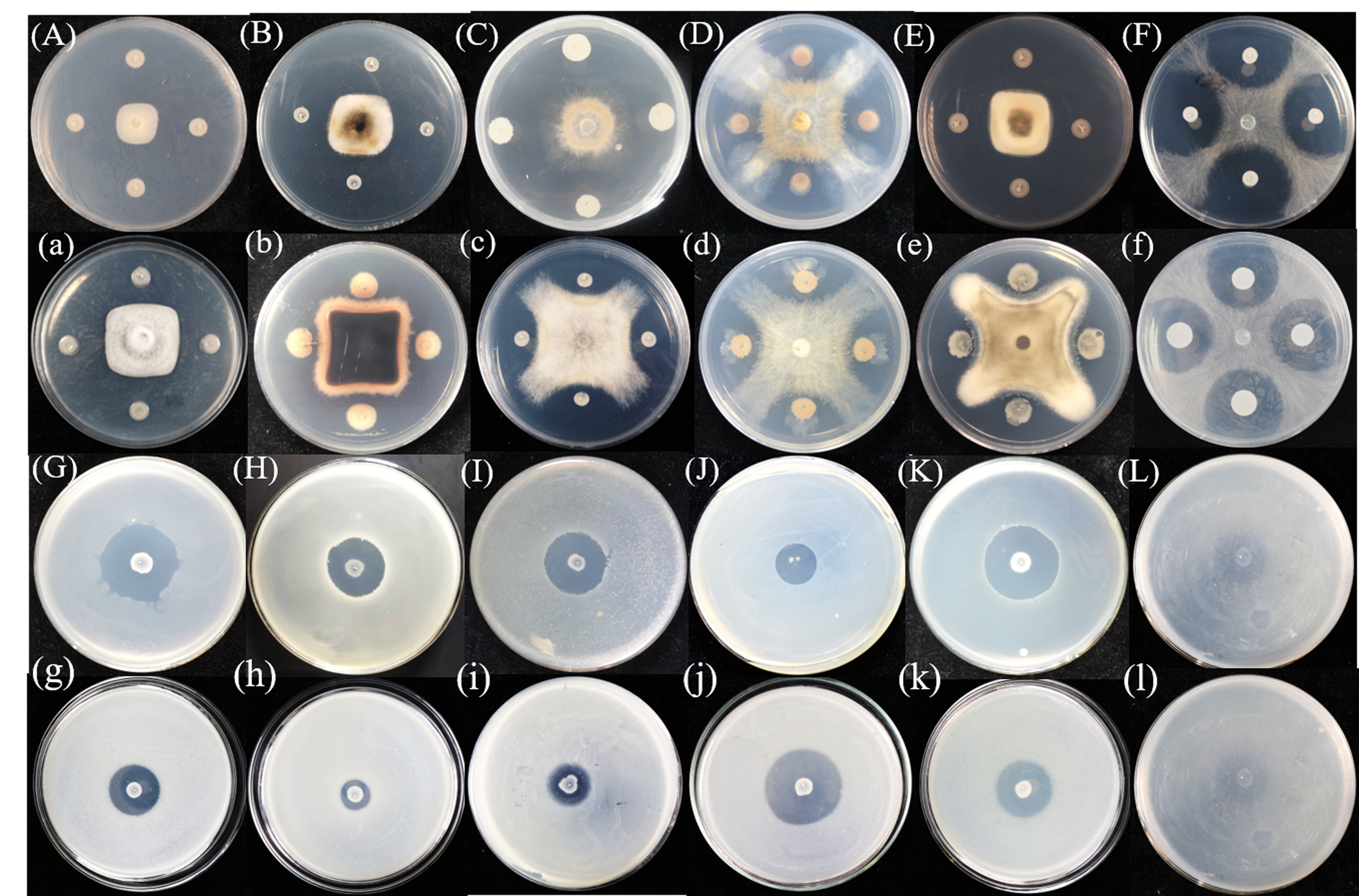

Supplement: Supplementary Figure 2 — Antagonistic assays of P. polymyxa ZF129 and ZF197 against various plant pathogens. (A,a) Verticillium dahlia, (B,b) Corynespora cassiicola, (C,c) Botrytis cinereal, (D,d) Fusarium oxysporum, (E,e) Colletotrichum spp., (F,f) Rhizoctonia solani, (G,g) Xanthomonas campestris pv. campestris, (H,h) Clavibacter michiganensis subsp. sepedonicum, (I,i) Ralstonia solanacearum, (J,j) Pseudomonas syringae pv. tomato, (K,k) Pseudomonas syringae pv. lachrymans, (L,l) control. [file Image_2.TIF]

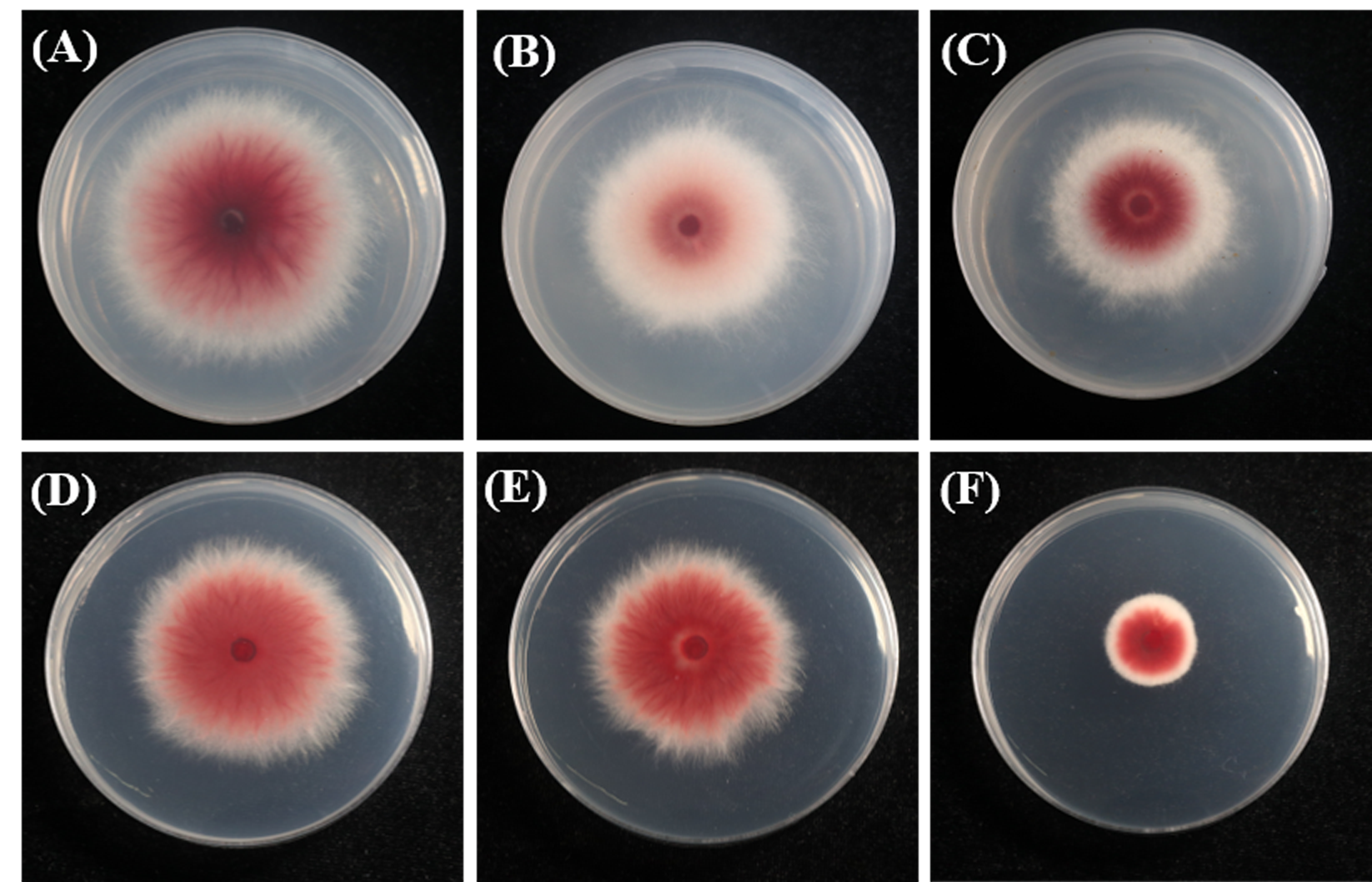

Supplement: Supplementary Figure 3 — Inhibitory activities of the volatiles or cell-free supernatants of P. polymyxa ZF129 and ZF197. (A) LB plate. (B) Volatiles of ZF129. (C) Volatiles of ZF197. (D) LB medium. (E) Cell-free supernatant of ZF129. (F) Cell-free supernatant of ZF197. [file Image_3.TIF]

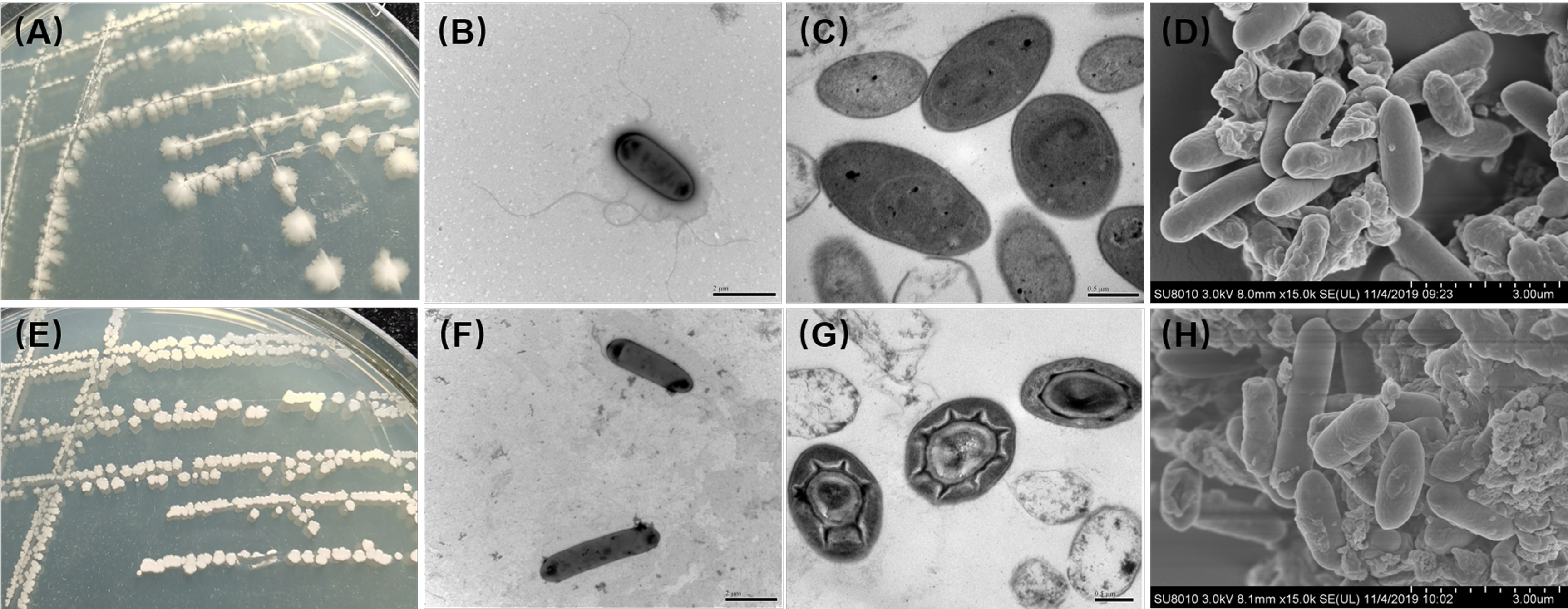

Supplement: Supplementary Figure 4 — General characteristics of P. polymyxa ZF129 and ZF197. (A,E) Images of ZF129 and ZF197 colony morphology. (B,F) images of ZF129 and ZF197 cells obtained using transmission electron microscopy. (C,G) images of ZF129 and ZF197 cell sections obtained using transmission electron microscopy (Hitachi 7700, Japan), (D,H) images of ZF129 and ZF197 cells obtained using scanning electron microscopy (Hitachi SU8010, Japan). [file Image_4.TIF]

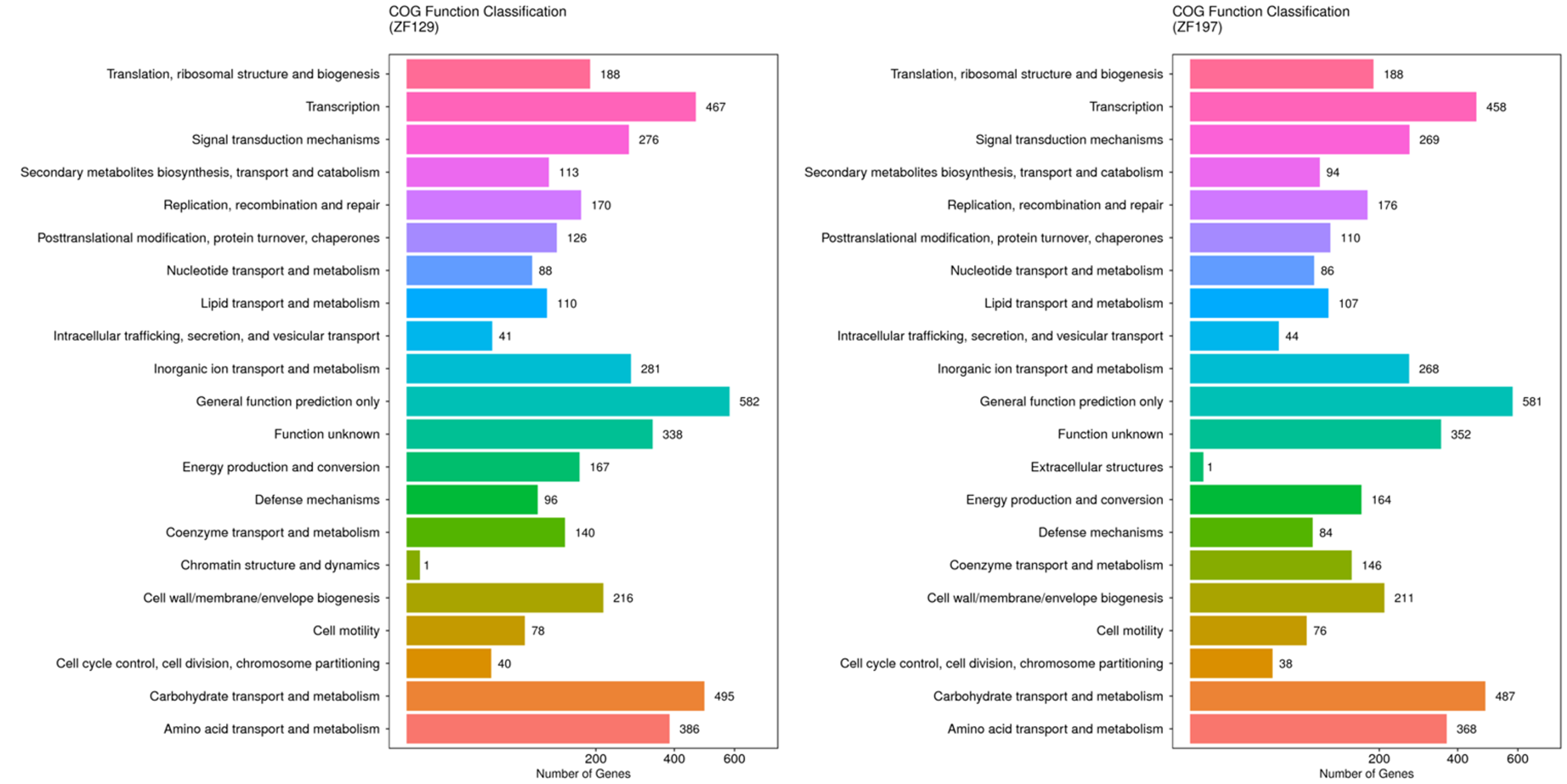

Supplement: Supplementary Figure 5 — COG functional categorization of two sequenced P. polymyxa genomes. COG functional categorization was performed using tools available in the Clusters of Orthologous Groups of proteins database. The left panel indicates the COG functional classification of ZF129, and the right panel indicates the COG functional classification of ZF197. [file Image_5.TIF]
