# Supplementary material for: Comparative and Functional Analyses of Two Sequenced Paenibacillus polymyxa Genomes Provides Insights Into Their Potential Genes Related to Plant Growth-Promoting Features and Biocontrol Mechanisms
Source: Front Genet. 2020 Dec 17;11:564939. doi: 10.3389/fgene.2020.564939 (PMC7773762; doi:10.3389/fgene.2020.564939)
Supplement: Supplementary Table 1 — The genome was assembled using the HGAP assembly pipeline in this study. [file Table_1.DOCX]

**TABLE S1** The genome was assembled using the HGAP assembly pipeline in this study.

| **[job.defaults]** | **#### Input** | **#### Data Partitioning** | **####Pre-assembly** | **####Pread overlapping** | **####Final Assembly** |
| --- | --- | --- | --- | --- | --- |
| njobs = 100  submit = qsub -S /bin/bash -sync y -V \  -q ${JOB_QUEUE} \  -N ${JOB_NAME} \  -o "${JOB_STDOUT}" \  -e "${JOB_STDERR}" \  -pe smp ${NPROC} \  "${JOB_SCRIPT}"  JOB_QUEUE = 16  MB = 30000  NPROC = 16 | [General]  input_fofn=input.fofn  input_type=raw  pa_DBdust_option=  pa_fasta_filter_option=pass  target=assembly  skip_checks=False  LA4Falcon_preload=false | pa_DBsplit_option=-x500 -s50  ovlp_DBsplit_option=-x500 -s50 | genome_size= 6000000  seed_coverage=30  length_cutoff=-1  pa_HPCdaligner_option=-v -B128 -t16 -M24  pa_daligner_option=-e.7 -l1000 -k18 -h80 -w8 -s100  falcon_sense_option=--output-multi --min-idt 0.70 --min-cov 2 --max-n-read 800  falcon_sense_greedy=False | ovlp_daligner_option=-e.96 -l2000 -k24 -h1024 -w6 -s100  ovlp_HPCdaligner_option=-v -B128 -M24 | overlap_filtering_setting=--max-diff 100 --max-cov 300 --min-cov 2  fc_ovlp_to_graph_option=  length_cutoff_pr=2000 |

The genome was assembled using the HGAP assembly pipeline in the pbsmrtpipe tool starting from subreads. The pbsmrtpipe tool is the secondary analysis workflow engine of Pacific Biosciences’ SMRT Analysis software v6.0.0.

**Software:**

SMRT Analysis software v6.0.0

FALCON
