# Supplementary material for: Comparative and Functional Analyses of Two Sequenced Paenibacillus polymyxa Genomes Provides Insights Into Their Potential Genes Related to Plant Growth-Promoting Features and Biocontrol Mechanisms
Source: Front Genet. 2020 Dec 17;11:564939. doi: 10.3389/fgene.2020.564939 (PMC7773762; doi:10.3389/fgene.2020.564939)
Supplement: Supplementary Table 2 — The locus tag information of 16S rRNA genes and five housekeeping genes used for phylogenetic tree construction in this study. [file Table_2.DOCX]

**TABLE S2** The locus tag information of 16S rRNA genes and five housekeeping genes used for phylogenetic tree construction in this study.

| **Strain (Name)** | **16S rRNA**  **16S ribosomal RNA** | ***gap*A**  **Glyceraldehyde-3-phosphate dehydrogenase** | ***gyr*A**  **DNA gyrase subunit A** | ***atp*D**  **ATP synthase subunit beta** | ***rpo*A**  **DNA-directed RNA polymerase subunit alpha** | ***rho***  **Transcription termination factor Rho** |
| --- | --- | --- | --- | --- | --- | --- |
| *Paenibacillus polymyxa* ZF129 | FGY93_04045 | FGY93_07260 | FGY93_08195 | FGY93_10565 | FGY93_11500 | FGY93_07510 |
| *Paenibacillus polymyxa* ZF197 | FQU75_02795 | FQU75_17710 | FQU75_05735 | FQU75_14340 | FQU75_13390 | FQU75_17460 |
| *Paenibacillus polymyxa* HY96-2 | C1A50_0092 | C1A50_0196 | C1A50_0009 | C1A50_4826 | C1A50_4624 | C1A50_0147 |
| *Paenibacillus polymyxa* SQR-21 | PPSQR21_038900 | PPSQR21_001880 | PPSQR21_000080 | PPSQR21_047030 | PPSQR21_045080 | PPSQR21_001380 |
| *Paenibacillus polymyxa* Sb3-1 | RE92_RS11270 | RE92_RS10755 | RE92_RS11655 | RE92_RS14015 | RE92_RS14960 | RE92_RS11000 |
| *Paenibacillus polymyxa* SC2 | PPSC2_00050 | PPSC2_c0186 | PPSC2_00040 | PPSC2_23245 | PPSC2_22295 | PPSC2_00705 |
| *Paenibacillus polymyxa* M1 | PPM_r001 | PPM_0170 | PPM_0008 | PPM_4616 | PPM_4426 | PPM_0135 |
| *Paenibacillus polymyxa* J | AOU00_09710 | AOU00_13740 | AOU00_01680 | AOU00_10360 | AOU00_09385 | AOU00_13490 |
| *Paenibacillus polymyxa* E681 | PPE_00749 | PPE_00195 | PPE_00008 | PPE_04454 | PPE_04250 | PPE_00139 |
| *Paenibacillus polymyxa* CR1 | X809_00055 | X809_00635 | X809_00045 | X809_39780 | X809_38785 | X809_00925 |
| *Paenibacillus polymyxa* YC0573 | PPYC2_24835 | PPYC2_25715 | PPYC2_26640 | PPYC2_02495 | PPYC2_03495 | PPYC2_25965 |
| *Paenibacillus polymyxa* YC0136 | PPYC1_23820 | PPYC1_23280 | PPYC1_24205 | PPYC1_02395 | PPYC1_03290 | PPYC1_23530 |
| *Paenibacillus polymyxa* ATCC 15970 | VK72_00050 | VK72_00970 | VK72_00040 | VK72_24140 | VK72_23130 | VK72_00725 |
| *Bacillus amyloliquefaciens* DSM 7 | BAMF_RS20260 | BAMF_3256 | BAMF_0007 | BAMF_3518 | BAMF_0143 | BAMF_3545 |
| *Bacillus amyloliquefaciens* TA208 | BAMTA208_r20829 | BAMTA208_17285 | BAMTA208_00035 | BAMTA208_18645 | BAMTA208_00720 | BAMTA208_18790 |
| *Bacillus velezensis* FZB42 | RBAM_000080 | RBAM_031300 | RBAM_000070 | RBAM_033970 | RBAM_001680 | RBAM_034240 |
| *Bacillus velezensis* SQR9 | V529_r00010 | V529_33910 | V529_00070 | V529_36660 | V529_01460 | V529_36930 |
| *Bacillus subtilis* 168 | BSU_rRNA_1 | BSU_33940 | BSU_00070 | BSU_36810 | BSU_01430 | BSU_37080 |
| *Bacillus subtilis* BSn5 | BSn5_r21080 | BSn5_05340 | BSn5_11590 | BSn5_09355 | BSn5_12295 | BSn5_09490 |
| *Bacillus subtilis* XF-1 | C663_r65 | C663_3274 | C663_0008 | C663_3580 | C663_0142 | C663_3607 |
| *Bacillus licheniformis* DSM 13 | BLi00008 | BLi03665 | BLi00007 | BLi03926 | BLi00161 | BLi03956 |
| *Bacillus licheniformis* ATCC 9789 | CPQ91_00040 | CPQ91_15615 | CPQ91_00035 | CPQ91_19890 | CPQ91_00885 | CPQ91_20045 |
| *Bacillus megaterium* DSM 319 | BMD_0007 | BMD_4746 | BMD_0006 | BMD_5134 | BMD_0160 | BMD_5158 |
| *Bacillus megaterium* ATCC 14581 | BG04_2283 | BG04_1751 | BG04_2282 | BG04_2142 | BG04_2436 | BG04_2166 |
| *Bacillus pumilus* TUAT1 | BTUAT1_r00010 | BTUAT1_31300 | BTUAT1_00070 | BTUAT1_33780 | BTUAT1_01480 | BTUAT1_34030 |
| *Bacillus pumilus* SH-B11 | UP15_00040 | UP15_13545 | UP15_00035 | UP15_17320 | UP15_00870 | UP15_17445 |

**Abbreviation:** ZF129: *Paenibacillus polymyxa* ZF129; HY96-2: *Paenibacillus polymyxa* HY96-2; SQR-21: *Paenibacillus polymyxa* SQR-21; Sb3-1: *Paenibacillus polymyxa* Sb3-1; SC2: *Paenibacillus polymyxa* SC2; M1: *Paenibacillus polymyxa* M1; J: *Paenibacillus polymyxa* J; E681: *Paenibacillus polymyxa* E681; YC0573: *Paenibacillus polymyxa* YC0573; YC0136: *Paenibacillus polymyxa* YC0136; ATCC 15970: *Paenibacillus polymyxa* ATCC 15970; DSM 7: *Bacillus amyloliquefaciens* DSM 7; TA208: *Bacillus amyloliquefaciens* TA208; FZB42: *Bacillus velezensis* FZB42; SQR9: *Bacillus velezensis* SQR9; 168: *Bacillus subtilis* 168; BSn5: *Bacillus subtilis* BSn5; XF-1: *Bacillus subtilis* XF-1; DSM 13: *Bacillus licheniformis* DSM 13; ATCC 9789: *Bacillus licheniformis* ATCC 9789; DSM 319: *Bacillus megaterium* DSM 319; ATCC 14581: *Bacillus megaterium* ATCC 14581; TUAT1: *Bacillus pumilus* TUAT1; SH-B11: *Bacillus pumilus* SH-B11.
