# Supplementary material for: Comparative and Functional Analyses of Two Sequenced Paenibacillus polymyxa Genomes Provides Insights Into Their Potential Genes Related to Plant Growth-Promoting Features and Biocontrol Mechanisms
Source: Front Genet. 2020 Dec 17;11:564939. doi: 10.3389/fgene.2020.564939 (PMC7773762; doi:10.3389/fgene.2020.564939)
Supplement: Supplementary Table 3 — Inhibition rates of 27 biocontrol bacteria against F. oxysporum. [file Table_3.DOCX]

**TABLE S3** Inhibition rate of twenty-seven biocontrol bacteria on *F. oxysporum*.

| No. | Strain species | Antifungal diameter (cm) | Inhibitory rate/% |
| --- | --- | --- | --- |
| ZF90 | *Bacillus subtilis* | 3.57±0.12 m | 54.27±1.48 b |
| ZF93 | *Bacillus amyloliquefaciens* | 3.60±0.17 m | 53.85±2.22 b |
| ZF100 | *Bacillus velezensis* | 3.97±0.06 l | 49.15±0.74 c |
| ZF103 | *Bacillus velezensis* | 5.73±0.21 e | 26.50±2.67 j |
| ZF113 | *Bacillus velezensis* | 5.97±0.12 d | 23.50±1.48 k |
| ZF114 | *Bacillus subtilis* | 3.83±0.06 l | 50.85±0.74 c |
| ZF115 | *Bacillus subtilis* | 4.60±0.10 k | 41.03±1.28 d |
| ZF118 | *Bacillus subtilis* | 5.07±0.06 gh | 35.04±0.74 gh |
| ZF122 | *Bacillus velezensis* | 5.87±0.12 de | 24.79±1.48 jk |
| ZF129 | *Paenibacillus polymyxa* | 3.10±0.10 n | 60.26±1.28 a |
| ZF132 | *Bacillus velezensis* | 7.03±0.12 a | 9.83±1.48 n |
| ZF138 | *Bacillus pumilus* | 5.33±0.15 f | 31.62±1.96 i |
| ZF141 | *Bacillus vallismortis* | 4.67±0.15 jk | 40.17±1.96 de |
| ZF145 | *Bacillus amyloliquefaciens* | 3.53±0.06 m | 54.70±0.74 b |
| ZF147 | *Bacillus velezensis* | 5.43±0.21 f | 30.34±2.67 i |
| ZF153 | *Bacillus tequilensis* | 4.87±0.06 hij | 37.61±0.74 efg |
| ZF158 | *Bacillus amyloliquefaciens* | 4.97±0.15 ghi | 36.32±1.96 fgh |
| ZF168 | *Bacillus subtilis* | 3.87±0.06 l | 50.43±0.74 c |
| ZF171 | *Bacillus subtilis* | 4.77±0.12 ijk | 38.89±1.48 def |
| ZF179 | *Bacillus tequilensis* | 6.23±0.12 c | 20.09±1.48 l |
| ZF183 | *Bacillus safensis* | 5.50±0.10 f | 29.49±1.28 i |
| ZF187 | *Bacillus subtilis* | 6.67±0.15 b | 14.53±1.96 m |
| ZF190 | *Bacillus tequilensis* | 5.10±0.10 g | 34.62±1.28 h |
| ZF197 | *Paenibacillus polymyxa* | 3.13±0.12 n | 59.83±1.48 a |
| ZF201 | *Bacillus subtilis* | 4.67±0.12 jk | 40.17±1.48 de |
| ZF203 | *Bacillus pumilus* | 4.73±0.06 jk | 39.32±0.74 de |
| ZF207 | *Bacillus tequilensis* | 3.87±0.06 l | 50.43±0.74 c |
