# Supplementary material for: Comparative and Functional Analyses of Two Sequenced Paenibacillus polymyxa Genomes Provides Insights Into Their Potential Genes Related to Plant Growth-Promoting Features and Biocontrol Mechanisms
Source: Front Genet. 2020 Dec 17;11:564939. doi: 10.3389/fgene.2020.564939 (PMC7773762; doi:10.3389/fgene.2020.564939)
Supplement: Supplementary Table 4 — The effects of strains ZF129 and ZF197 against various plant pathogens. [file Table_4.DOCX]

**TABLE** **S4** The effects of strain ZF129 and ZF197 against various plant pathogens.

| Pathogens | Antimicrobial diameter of ZF129 (cm) | Inhibition rate of ZF129 (%) | Antimicrobial diameter of ZF197 (cm) | Inhibition rate of ZF197 (%) |
| --- | --- | --- | --- | --- |
| ***Fungi*** | | | | |
| *Verticillium dahliae* | - | 72.16±0.90 a | - | 41.56±1.44b |
| *Corynespora cassiicola* | - | 60.59±2.04 bc | - | 7.46±3.51e |
| *Botrytis cinerea* | - | 63.33±0.34 b | - | 34.13±2.30c |
| *Fusarium oxysporum* | - | 60.00±2.70 bc | - | 28.75±0.00d |
| *Colletotrichum* spp. | - | 54.71±2.35 d | - | 32.53±2.74c |
| *Rhizoctonia solani* | - | 55.29±1.56 d | - | 69.65±2.23a |
| ***Bacteria*** | | | | |
| *Xanthomonas campestris*pv. *campestris* | 3.40±2.58 a | - | 2.32±0.04b | - |
| *Clavibacter michiganensis* subsp. *sepedonicum* | 2.12±0.69 c | - | 1.31±0.01d | - |
| *Ralstonia solanacearum* | 2.30±2.08 c | - | 1.71±0.09c | - |
| *Pseudomonas syringae* pv. *tomato* | 1.78±0.69 d | - | 3.35±0.03a | - |
| *Pseudomonas syringae* pv. *lachrymans* | 2.78±0.69 b | - | 2.40±0.04b | - |
| Control | - | - | - | - |
