# Supplementary material for: Comparative and Functional Analyses of Two Sequenced Paenibacillus polymyxa Genomes Provides Insights Into Their Potential Genes Related to Plant Growth-Promoting Features and Biocontrol Mechanisms
Source: Front Genet. 2020 Dec 17;11:564939. doi: 10.3389/fgene.2020.564939 (PMC7773762; doi:10.3389/fgene.2020.564939)
Supplement: Supplementary Table 5 — Inhibitory activities of the volatiles or cell-free supernatants of P. polymyxa ZF129 and ZF197. [file Table_5.DOCX]

**TABLE S5** Inhibitory activity of the volatiles or cell-free supernatant of *Paenibacillus polymyxa* ZF129 and ZF197.

| Treatment | Colony diameter (cm) | Inhibitory activity (%) |
| --- | --- | --- |
| LB plate | 6.05±0.29 a | - |
| volatiles of ZF129 | 4.73±0.09 b | 21.82 |
| volaties of ZF197 | 4.47±0.09 c | 26.12 |
| LB medium | 6.47±0.05 a | - |
| Cell-free Supernatant of ZF129 | 5.97±0.07 b | 7.73 |
| Cell-free Supernatant of ZF197 | 2.80±0.08 c | 56.72 |
