# Supplementary material for: Comparative and Functional Analyses of Two Sequenced Paenibacillus polymyxa Genomes Provides Insights Into Their Potential Genes Related to Plant Growth-Promoting Features and Biocontrol Mechanisms
Source: Front Genet. 2020 Dec 17;11:564939. doi: 10.3389/fgene.2020.564939 (PMC7773762; doi:10.3389/fgene.2020.564939)
Supplement: Supplementary Table 6 — Genome statistics. [file Table_6.DOCX]

**TABLE S6** Genome statistics.

| Attribute | ZF129 Value | ZF197 Value |
| --- | --- | --- |
| Genome size (bp) | 5,820,553 | 5,539,234 |
| Plasmid size (bp) | 116,622 | 32,065 |
| DNA coding (bp) | 4,980,873 | 4,703,361 |
| GC content (%) | 45.34 | 45.60 |
| DNA scaffolds | 3 | 2 |
| Contigs | 3 | 2 |
| Predicted genes | 5,149 | 5,054 |
| Protein coding genes | 4,861 | 4,902 |
| RNA genes | 156 | 152 |
| Pseudo genes | 132 | 244 |
| Genes in internal clusters | ^a^ND | ^a^ND |
| Genes with function prediction | 4399 | 4308 |
| Genes assigned to COGs | 3,740 | 3,717 |
| Genes assigned to GOs | 3,424 | 3,429 |
| Genes assigned to Swissprots | 3,753 | 3,696 |
| CRISPR repeats | ^a^ND | 3 |

^a^ND = not determined.
